# The Added Value of Different Data Types for Calibrating and Testing a Hydrologic Model in a Small Catchment

**DOI:** 10.1029/2019WR026153

**Published:** 2020-10-08

**Authors:** B. Széles, J. Parajka, P. Hogan, R. Silasari, L. Pavlin, P. Strauss, G. Blöschl

**Affiliations:** ^1^ Institute of Hydraulic Engineering and Water Resources Management Vienna University of Technology Vienna Austria; ^2^ Federal Agency of Water Management Institute for Land and Water Management Research Petzenkirchen Austria

**Keywords:** hydrologic model, model parameterization, field observations, experimental catchment

## Abstract

This study investigated the added value of different data for calibrating a runoff model for small basins. The analysis was performed in the 66 ha Hydrological Open Air Laboratory, in Austria. An Hydrologiska Byråns Vattenbalansavdelning (HBV) type, spatially lumped hydrologic model was parameterized following two approaches. First, the model was calibrated using only runoff data. Second, a step‐by‐step approach was followed, where the modules of the model (snow, soil moisture, and runoff generation) were calibrated using measurements of runoff and model state variables and output fluxes. These measurements comprised laser‐based measurements of precipitation, satellite and camera observations of snow, ultrasonic measurements of snow depth, eddy covariance measurements of evapotranspiration, time domain transmissometry‐based soil moisture measurements, time‐lapse photography of overland flow, and groundwater level measurements by piezometers. The two model parameterizations were evaluated on annual, seasonal, and daily time scales, in terms of how well they simulated snow, soil moisture, evapotranspiration, overland flow, storage change in the saturated zone, and runoff. Using the proposed step‐by‐step approach, the relative runoff volume errors in the calibration and validation periods were 0.00 and −0.01, the monthly Pearson correlation coefficients were 0.92 and 0.82, and the daily logarithmic Nash Sutcliffe efficiencies were 0.59 and 0.18, respectively. By using different sources of data besides runoff, the overall process consistency improved, compared to the case when only runoff was used for calibration. Soil moisture and evapotranspiration observations had the largest influence on simulated runoff, while the parameterization of the snow and runoff generation modules had a smaller influence.

## Key Points

- A new framework is presented for stepwise runoff model parameter estimation from observed runoff and model state variables and fluxes
- For the study catchment, correlation coefficient of monthly runoff in the validation period is 0.82, and the relative volume error is −1%
- Combination of soil moisture and evapotranspiration observations had the largest influence on parameter estimation

## 1 Introduction

Observed runoff represents the overall, aggregated catchment behavior. Therefore, runoff observations are the most common information used for identifying the parameters of hydrologic models. However, parameters of conceptual and physically based hydrologic models usually cannot be identified accurately using observed runoff alone as it is difficult to decide, whether the model performs well for the right reasons (Beven & Freer, 2001; Savenije, 2001; Viglione et al., 2018). One way of dealing with this issue is to use additional measurements of input and output fluxes and model states in hydrologic modeling. Additional information on hydrologic processes helps to constrain and validate hydrologic models and testing whether they get the right answers for the right reasons (Grayson et al., 1992).

Most of the studies use other measurements of fluxes and states in multiple objective calibration as a part of the objective function. Previous studies used ground‐based or alternatively remote sensing products or their combination as such additional information on hydrologic processes. Soil moisture (Kundu et al., 2017; Kunnath‐Poovakka et al., 2016; Parajka et al., 2006; Rajib et al., 2016; Shahrban et al., 2018), evapotranspiration (Gui et al., 2019; Herman et al., 2018; Immerzeel & Droogers, 2008; Kunnath‐Poovakka et al., 2016), and groundwater level data (Demirel et al., 2019; Seibert, 2000) were often used for model calibration to improve the model's internal consistency. These studies showed the added value of different observations besides runoff, for example, for soil moisture, evapotranspiration, and groundwater levels. But only a few studies combined the different type of observations (e.g., Avanzi et al., 2020; Kuras et al., 2011). In the past, these data were used mainly in connection with calibration of physically based hydrologic models, where measurements could be more explicitly linked to the simulations than for conceptual hydrologic models. The performance of the distributed hydrology soil vegetation model was evaluated by Thyer et al. (2004) and Kuras et al. (2011) using field data. Their study area was located in British Columbia, Canada. While Thyer et al. (2004) focused mainly only on the micrometeorological part of the process‐based model (such as snowmelt and energy balance) and also used observed hydrograph from another, nearby catchment, Kuras et al. (2011) also evaluated the subsurface and surface runoff dynamics with a spatially extensive database. Thyer et al. (2004) and Kuras et al. (2011) achieved a daily Nash Sutcliffe efficiency for runoff of approximately 0.90, and Thyer et al. (2004) stated that runoff simulations were most sensitive to snowmelt characteristics as runoff was driven by spring snowmelt in their high elevated, forested study region. In another study, Wei et al. (2016) used measurements of snow water equivalent, snow depth, transpiration, stomatal feedback to vapor pressure, soil and forest properties, and soil moisture to parameterize a physically based model without a flow routing module to simulate the water balance in a 4 km^2^ catchment in the United States. Without using runoff data for model calibration, they could reproduce the annual and monthly variabilities of potential runoff (combined outputs of surface runoff and deep drainage) with a Nash Sutcliffe efficiency of 0.62 and 0.56, respectively. A similar study was performed by Kuppel et al. (2018) in the Scottish Highlands with a fully distributed ecohydrological model. They could simulate daily runoff reasonably well without using runoff observations, but the model performance was substantially better, when runoff was also included in the calibration. Kuppel et al. (2018) argued that the spatiotemporal footprint of the observations involved in model calibration had to be carefully considered. They found that certain variables could only be well simulated, when the model was calibrated to measurements of that variable, for instance, soil moisture in gley soils and transpiration in Scots pine stands.

When ground‐based monitoring data are not available, remote sensing products may be a useful alternative for parameter estimation (López et al., 2017; Nijzink et al., 2018; Silvestro et al., 2015). López et al. (2017) found on a Moroccan catchment with Mediterranean and semiarid climate that runoff could be better estimated when both remotely sensed evapotranspiration and remotely sensed soil moisture products were involved in calibrating a large‐scale hydrologic model compared to a scenario, when these products were used independently. Silvestro et al. (2015) also found that using data from both ground stations and remotely sensed products, that is, land surface temperature and surface soil moisture estimates, improved the models internal consistency in two Italian catchments with temperate climates. Nijzink et al. (2018) comprehensively tested nine remotely sensed products on 27 European catchments with diverse landscapes and climates. The products included remotely sensed soil moisture, evaporation, total water storage, and snow accumulation. They found that two soil moisture products, the Gravity Recovery and Climate Experiment (GRACE) total water storage anomalies, and in snow‐dominated catchments the Moderate Resolution Imaging Spectroradiometer (MODIS) snow cover products helped the most in constraining model parameters, when runoff data were not available. Remotely sensed surface water extent and water levels have also been found to be useful proxies on large river basins (Liu et al., 2015; Revilla‐Romero et al., 2015; Sun et al., 2012, 2015), while Corbari et al. (2015) used satellite land surface temperature data for distributed hydrologic model calibration. Ruiz‐Perez et al. (2017) used only MODIS Normalized Difference Vegetation Index (NDVI) data to calibrate an ecohydrological model and obtained good runoff estimates at the catchment outlet. The resolution of these remote sensing products, both in time and space, is usually too coarse for small catchments. Therefore, small catchment scale processes require field observations (e.g., Avanzi et al., 2020). However, there are only a few extensively monitored research catchments in the world, where such long‐term field observations are available.

An alternative is to use stepwise calibration. Most of the studies used runoff signatures, for example, low flows, high flows, annual runoff, and so forth to calibrate their models step‐by‐step (e.g., Fenicia et al., 2007; Gelleszun et al., 2017; Hogue et al., 2000; Lu & Li, 2015). For instance, Hogue et al. (2000) separated the parameterization of the low and high flow simulations. Gelleszun et al. (2017) separately calibrated the parameters influencing runoff volume and peaks, seasonality and low flows, and the shape of the hydrograph. Fenicia et al. (2007) compared two multiobjective model calibration approaches. One of these approaches was a stepped calibration approach, where they separately calibrated certain parameter sets associated with different processes. These processes influenced distinct aspects of the system response, low flows, high flows, and lag time. Lu and Li (2015) proposed a different calibration strategy, grouping the model parameters according to time scales (annual, seasonal, and daily) where they are the most sensitive. Only a few studies used measurements on different fluxes and states in a stepwise fashion. These studies performed stepwise model calibration by looking at the internal state variables and processes of the model (e.g., Arheimer et al., 2020; Avanzi et al., 2020; Hay et al., 2006; Kuras et al., 2011; Ning et al., 2015). Hay et al. (2006) calibrated solar radiation, potential evapotranspiration, water balance, and daily runoff using measurements of fluxes and states and runoff, while Ning et al. (2015) calibrated the water storage and runoff generation. Inspired by these studies, which used stepwise model calibration approaches, in this study we aimed to link model simulations with field observations focusing on the different hydrologic processes. We used a large set of field observations of input and output fluxes and states besides runoff to calibrate our model in a step‐by‐step way.

The objective of this study was to investigate the added value of different data types of hydrologic processes for calibrating and testing a hydrologic model in a small catchment. Our hypothesis was that, by using additional data apart from runoff for calibrating a lumped conceptual hydrologic model, process consistency would improve. Our goal was to propose a stepwise approach for constraining hydrologic model by using runoff data and observations of snow, soil moisture, evapotranspiration, overland flow, and groundwater levels. We aimed at linking field observations with lumped, conceptual hydrologic model simulations by using all the available data in a stepwise mode. The model performance was evaluated at the annual, seasonal, and daily time scales. The analysis was performed in the Hydrological Open Air Laboratory (HOAL) in Austria, a 66 ha experimental catchment, where a large variety of long‐term field observations are available (Blöschl et al., 2016).

## 2 Study Area and Data

### 2.1 Study Area

**Figure 1:**
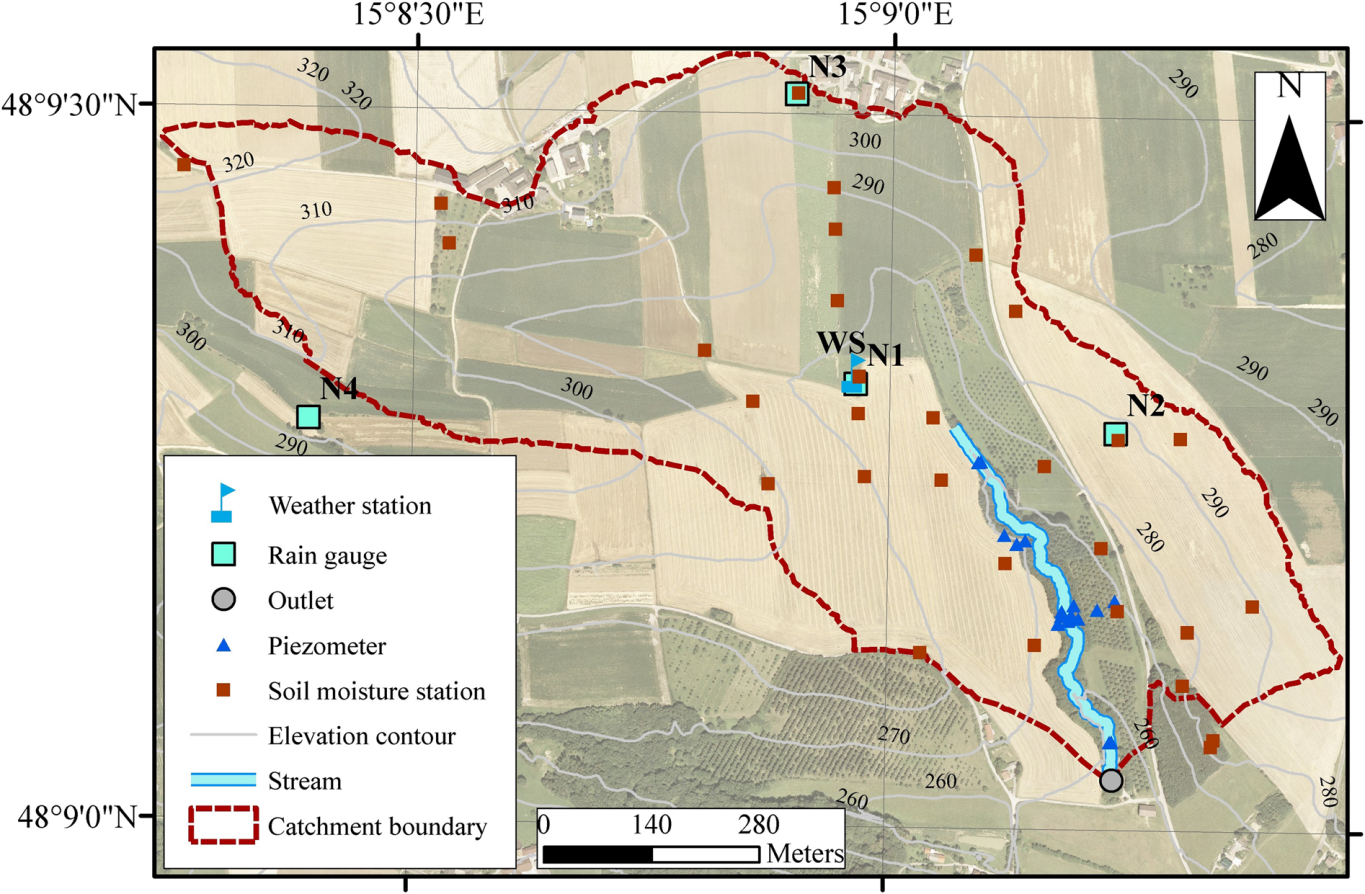
Study area: Hydrological Open Air Laboratory (HOAL) in Petzenkirchen, Lower Austria, and location of devices for precipitation and evapotranspiration (weather station), soil moisture, and groundwater level (piezometer) measurements.

The study was conducted in a small experimental catchment, the Hydrological Open Air Laboratory (HOAL) in Petzenkirchen, located in the western part of Lower Austria (Figure 1), approximately 100 km west of Vienna (48° 9′ N, 15° 9′ E) (Blöschl et al., 2016). The drainage area of the catchment is 66 ha at the catchment outlet. The natural surface water outlet of the catchment is the Seitengraben stream. The elevation of the catchment ranges from 257 to 323 m above sea level, with a mean slope of 8%. The stream is approximately 620 m long and has a medium slope of 2.4% (Eder et al., 2010, 2014; Exner‐Kittridge et al., 2016; Széles et al., 2018).

The climate is humid. Mean annual (1991–2017) air temperature, precipitation, runoff and evapotranspiration, and storage change estimated from the water balance are 9.6°C, 782 mm/year, 184 mm/year, and about 598 mm/year, respectively. Seasonal maxima of air temperature, rainfall amount, and intensity occur in the summer. Mean monthly runoff tends to peak in winter or early spring. The amount of snow falling in winter is small and quickly melts in the catchment. On average (2013–2017), snow is observed on less than 10% of the days in a year. A significant amount of snow was only observed in 2016 and 2017 in the catchment, while the winters of 2014 and 2015 were almost snow‐free.

The geology of the HOAL consists of Tertiary fine sediments and fractured siltstone of the Molasse zone. The dominant soil types are Cambisols (57%), Kolluvisol (16%), and Planosols (21%) with moderate to low permeability. Gleysols (6%) occur close to the stream (Blöschl et al., 2016; FAO, ISRIC and ISSS, 1998).

The catchment is dominated by agricultural land use. Eighty‐seven percent of the catchment area is arable land; the rest is forested, paved, or used as pasture. Main crops are winter wheat, maize, winter barley, and winter oilseed rape.

### 2.2 Data

Rainfall has been recorded by a tipping bucket at 7, 14, and 19 hr between 1986 and 1991 situated approximately 700 m away from the catchment outlet, then by an automated weighing rain gauge (Kroneis Pesa) since 1991. Rainfall has been measured with high temporal resolution (1 min) by a weighing rain gauge (OTT Pluvio) situated 200 m from the catchment outlet since 2002. In 2012, four additional weighing rain gauges (OTT Pluvio) were installed in the HOAL. Since 2013, a laser‐based present weather sensor located at the weather station (Campbell PWS100) has measured the size and velocity of water droplets in the air with 1 min temporal resolution, and time‐lapse photographs have been taken by the weather station camera (Sanyo VCC‐MCH5600P) every minute during daylight. Since 1986, air temperature (Pt 1000) has been recorded at 7, 14, and 19 hr by a thermometer. Since October 2012, air temperature and relative humidity at 2 m height (HMP 155), air pressure (Campbell EC100), and snow depth (SR50AT) have been measured at the HOAL weather station with half hourly temporal resolution (Figure 1).

Twenty‐nine time domain transmission soil moisture stations, 19 permanent and 10 temporary stations, with sensors at 0.05, 0.10, 0.20, and 0.50 m depths have measured the water content in the unsaturated zone since 2013 with hourly temporal resolution. Since August 2012, grass evapotranspiration has been measured by a closed‐path eddy covariance station (Campbell EC155) at the weather station, and crop evapotranspiration has been measured by two open‐path eddy covariance stations (Campbell IRGASON) at various locations according to the agricultural crop rotation.

Nineteen piezometers (SWS Mini Driver) located in the riparian forest close to the stream have monitored the groundwater level at a resolution of 5 min since 2013.

Runoff has been monitored at the outlet of the catchment by a calibrated H‐flume with a pressure transducer since 2001 and additionally with an ultrasonic probe since mid‐2010 with 1 min temporal resolution (Figure 1). Details on the sensors are given in Blöschl et al. (2016).

**Figure 2:**
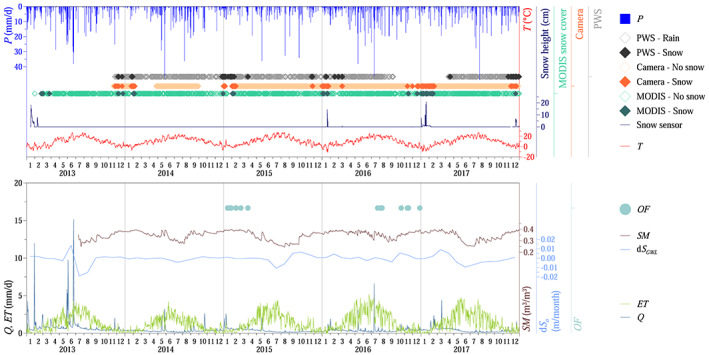
Field observations since 2013, used for model calibration and validation. (top) Air temperature *T*, precipitation *P* (average of four rain gauges), snow depth measured by snow sensor, reclassified categories from MODIS snow cover images, snow cover based on time‐lapse photos from the digital camera located at the weather station, and reclassified categories from present weather sensor (PWS). (bottom) Runoff *Q*, evapotranspiration *ET*, soil moisture *SM* (average of all stations over all depths), monthly average storage change in the saturated zone *dS*_*o*_ (catchment average of spatially interpolated raster map based on piezometers in the riparian zone), and occurrence of overland flow *OF*.

Three time periods were selected for the analysis, a 22 year long period when only runoff measurements (1991–2012) and a 3 year long (2013–2015) and a 2 year long (2016–2017) period when runoff measurements and additional sources of data were available (Figure 2). The 3 year long period was used for model calibration (Calib) and the 22 year long (Val1) and 2 year long (Val2) periods for model validation. One year proceeding each period was used as warm‐up period.

A significant amount of snow was observed only in 2016 and 2017 in the catchment, while the winters of 2014 and 2015 were almost snow‐free. Years 2014 and 2015 were exceptionally dry years, while 2013, 2016, and 2017 were more wet (Figure 2).

## 3 Methodology

### 3.1 Hydrologic Model

**Figure 3:**
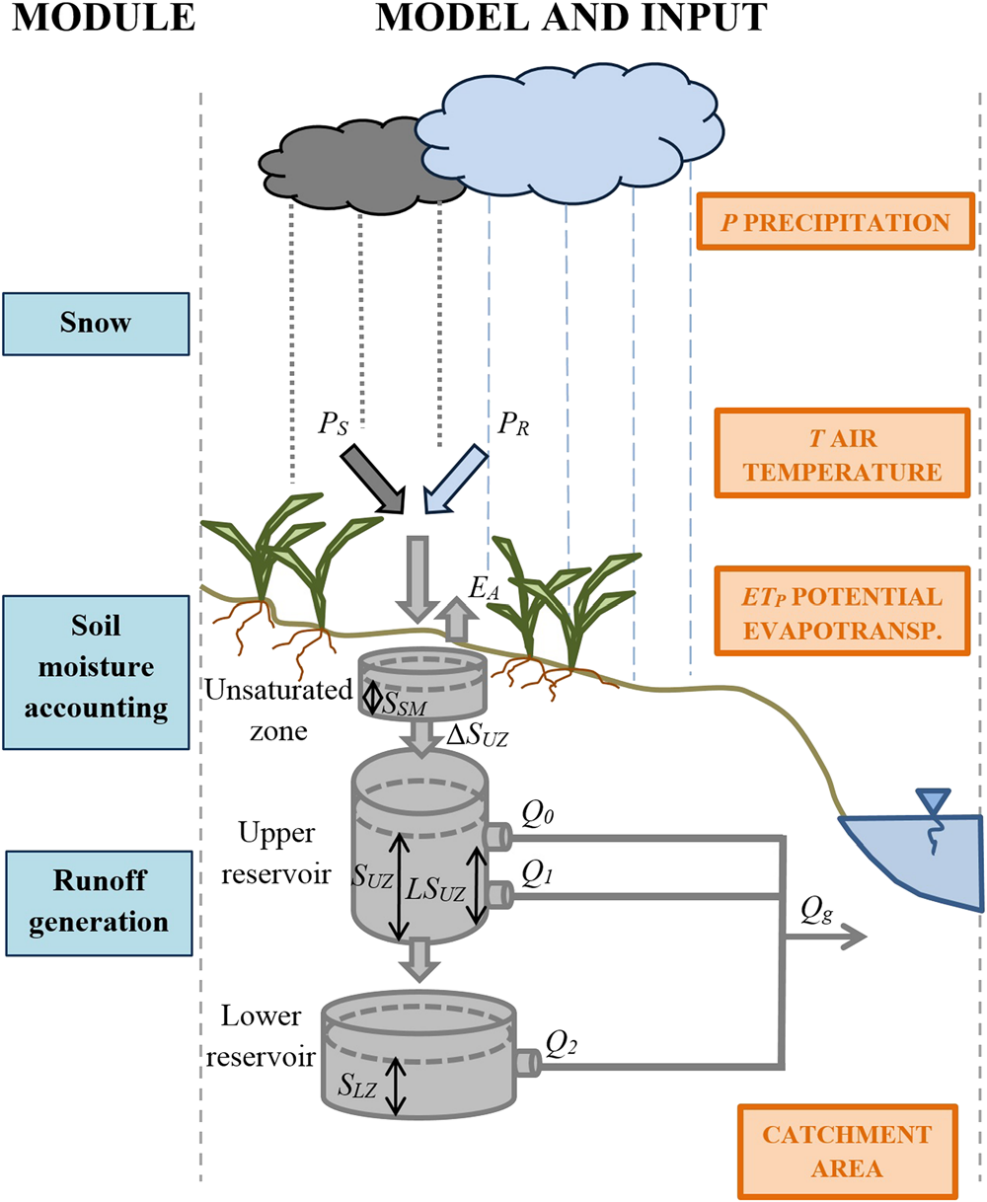
Model structure with the three modules (snow, soil moisture, and runoff generation) and input shown in orange rectangles on the right. Table 1 contains further information on the equations and notations.

A lumped, conceptual hydrologic model, the TUWmodel, was used in this study (Parajka et al., 2007). The model follows the structure of the Hydrologiska Byråns Vattenbalansavdelning (HBV) model (Bergström, 1976; Bergström & Lindström, 2015; Lindström et al., 1997). Numerous studies have shown that this type of model structure works well in Austrian catchments (e.g., Parajka et al., 2007; Sleziak et al., 2018) and worldwide (Bergström & Lindström, 2015). The model consists of three main modules: a snow module, a soil moisture accounting module, and a runoff generation module (Merz & Blöschl, 2004; Parajka et al., 2007) (Figure 3 and Table 1). It has 14 free model parameters, which need to be calibrated.

**Table 1:** Model Equations and Notations

Module	Equation		Notation
Snow module	Separation of solid and liquid precipitation:		Model input:
	*P_*S*_ = x · P*	(1)	‐*P* (mm/d) Precipitation
			‐*T* (°C) Air temperature

	*P*_*R*_ = (1 − *x*) · *P*	(2)	Model parameter:
	where:		‐*T*_*wb*_ (°C) Wet bulb temperature
	$\left\{\begin{array}{c} x=0\;\text{if}\;T_{\mathrm{wb}}\leq T\\ x=1\;\text{if}\;T<T_{\mathrm{wb}} \end{array}\right)$	(3)	‐*T*_*m*_ (°C) Threshold temperature, above which melt starts
			‐*DDF* (mm/°C/d) Degree day factor
			‐*SCF* (−) Snow correction factor
	Snow melt:		
	*P*_*m*_ = (*T* − *T*_*m*_) · *DDF if T*_*m*_ < *T and* 0 < *SWE*	(4)	State variable:
	Otherwise *P*_*m*_ = 0		‐*SWE*_*i*_ (mm) Snow water equivalent at time step *i*
		
	Snow storage:		Model output:
	*SWE*_*i*_ = *SWE*_*i* −_ _*1*_ + (*SCF* · *P*_*S*_ − *P*_*m*_) · Δ*t*	(5)	‐*P*_*S*_ (mm/d) Solid precipitation
			‐*P*_*R*_ (mm/d) Liquid precipitation
			*‐P*_*m*_ (mm/d) Snow melt

			Other:
			‐Δ*t* (d) Time step

Soil moisture accounting module	Change in soil moisture:		Model input:
	*S*_*SM*,*i*_ = *S*_*SM*,*i* − *1*_ + *P*_*R*_ + *P*_*m*_ − *ET* − Δ*S*_*UZ*_	(6)	‐*ET*_*P*_ (mm/d) Potential evapotranspiration
		
	Fraction of precipitation generating runoff:		Model parameter:
	$\Delta S_{\mathrm{UZ}}={\left(\frac{S_{\mathrm{SM}}}{\mathrm{FC}}\right)}^{\beta }\cdot \left(P_{R}+P_{m}\right)$	(7)	‐*FC* (mm) Field capacity, maximum soil moisture storage
			‐*β* (−) Nonlinear parameter for runoff production
			‐*LPrat* (−) Parameter related to the limit for potential evapotranspiration
	Actual evapotranspiration:		
	$\begin{array}{l} \mathrm{ET}={\mathrm{ET}}_{P}\frac{S_{\mathrm{SM}}}{\text{LPrat}\cdot \mathrm{FC}}\;\text{if}\;S_{\mathrm{SM}}<\text{LPrat}\cdot \mathrm{FC}\\ \mathrm{ET}={\mathrm{ET}}_{P}\;\text{if}\;S_{\mathrm{SM}}\geq \text{LPrat}\cdot \mathrm{FC} \end{array}$	(8)	State variable:
			‐*S*_*SM,i*_ (mm) Soil moisture at time step *i*
			‐Δ*S*_*UZ*_ (mm) Fraction of precipitation generating runoff

			Model output:
			‐*ET* (mm/d) Actual evapotranspiration

Runoff generation module	Hillslope routing:		Model parameter:
	$\begin{array}{l} q_{0}=\left(S_{\mathrm{UZ}}-{\mathrm{LS}}_{\mathrm{UZ}}\right)\frac{\exp\left(-\frac{1}{k_{0}}\right)}{k_{0}}\;\text{if}\;{\mathrm{LS}}_{\mathrm{UZ}}\leq S_{\mathrm{UZ}}\\ \;q_{0}=0\;\text{otherwise} \end{array}$	(9)	‐*k*_*0*_ (d) Storage time for very fast response
			‐*k*_*1*_ (d) Storage time for fast response
			‐*k*_*2*_ (d) Storage time for slow response
			‐*LS*_*UZ*_ (mm) Threshold storage state, very fast runoff *q*_*0*_ starts, if it is exceeded
	$q_{1}=-c_{P}+\left(c_{P}+\frac{S_{\mathrm{UZ}}}{k_{1}}\right)\cdot \exp\left(-\frac{1}{k_{1}}\right)$	(10)	‐*c*_*P*_ (mm/d) Percolation rate
			‐*B*_*MAX*_ (d) Maximum base at low flows
			‐*c*_*R*_ (d^2^/mm) Free scaling parameter
	$q_{2}=c_{P}-\left(c_{P}-\frac{S_{\mathrm{LZ}}}{k_{2}}\right)\cdot \exp\left(-\frac{1}{k_{2}}\right)$	(11)	
	*Q*_*g*_ = *q*_*0*_ + *q*_*1*_ + *q*_*2*_	(12)	State variable:
	*S*_*UZ*,*i*_ = *S*_*UZ*,*i* − *1*_ + Δ*S*_*UZ*,*i*_ − *q*_*1*_ − *c*_*P*_	(13)	‐*S*_*UZ*_ (mm) Storage state in upper reservoir
	*S*_*LZ*,*i*_ = *S*_*LZ*,*i* − *1*_ − *q*_*2*_ + *c*_*P*_	(14)	‐*S*_*LZ*_ (mm) Storage state in lower reservoir
			‐*B*_*q*_ (d) Duration of convolution

	Routing in the river – transfer function:		Model output:
	$\begin{array}{l} B_{Q}=B_{\mathrm{MAX}}-c_{R}\cdot Q_{g}\;\text{if}\;\left(B_{\mathrm{MAX}}-c_{R}\cdot Q_{g}\right)\geq 1\\ B_{Q}=1\;\text{otherwise} \end{array}$	(15)	‐*q*_*0*_ (mm/d) Surface runoff
			‐*q*_*1*_ (mm/d) Subsurface runoff
			‐*q*_*2*_ (mm/d) Baseflow

While we could have modified the model structure to tailor it to the runoff processes in the HOAL, we chose not to do this. Instead, we used a more general model structure that could be used in a wider range of catchments.

Within the snow module according to (1)–(5) in Table 1, precipitation *P* (mm/day) is separated into *P*_*S*_ solid and *P*_*R*_ liquid precipitation depending on the wet bulb temperature parameter *T*_*wb*_ (°C) (Bergström, 1976; Blöschl et al., 1991; Jennings et al., 2018; Steinacker, 1983). The catch deficit of precipitation gauges during snowfall is corrected by a snow correction factor *SCF* (−). Snowmelt *M* (mm/day) is simulated based on the degree‐day concept, using a degree day factor *DDF* (mm/°C/day) and a melt temperature parameter *T*_*m*_ (°C).

Within the soil moisture module according to (6)–(8) in Table 1, the fraction of precipitation producing runoff and evapotranspiration is simulated as a function of the soil moisture state *SM* (mm) of the catchment. If the soil moisture storage exceeds a threshold parameter *FC* (mm), all rainfall and melt contribute to runoff. The characteristics of runoff production are controlled by the nonlinearity parameter *β* (−). If the soil moisture state exceeds the limit for potential evapotranspiration *LP* (mm), which is the product of *FC* and parameter *LPrat* (−), actual evapotranspiration *ET* (mm/day) reaches its potential rate *ET*_*P*_ (mm/day). The potential evapotranspiration *ET*_*P*_ was calculated with the modified Blaney‐Criddle method (Parajka et al., 2003; Schrödter, 1985).

Within the runoff generation module according to (9)–(15) in Table 1, an upper and a lower reservoirs represent hillslope routing. The proportion of rainfall and melt contributing to runoff enters the upper reservoir and leaves it through three paths. The first path is very fast runoff *q*_*0*_ (mm/day) with very fast storage time *k*_*0*_ (day), if a threshold of the storage state *LS*_*UZ*_ (mm) is exceeded in the upper reservoir. The other two paths are an outflow from the upper reservoir *q*_*1*_ (mm/day) with a fast storage time *k*_*1*_ (day) and percolation to the lower reservoir with a constant percolation rate *c*_*P*_ (mm/day). Water leaves the lower zone as baseflow *q*_*2*_ (mm/day) with a slow storage time *k*_*2*_ (day). The outflow from the two reservoirs is routed by a triangular transfer function representing runoff routing in the stream, where *B*_*MAX*_ (day) is the maximum base at low flows and *c*_*R*_ (day^2^/mm) is a free scaling parameter.

Following a sensitivity analysis (supporting information Text S1 and Table S1), the study proceeded along two modeling approaches. In the first approach, the model was calibrated in one step on a daily temporal resolution, using only runoff data in the objective function. In the second approach, the model was parametrized step‐by‐step using additional data besides runoff, starting with (1) snow accumulation and melt processes, (2) evapotranspiration and soil moisture changes, and (3) runoff generation and storage changes in the saturated zone. The abbreviations of the scenarios are listed in Table 2. The calibrated model parameters are found in Table 3 for each scenario. For simulating snow accumulation, a half‐hourly temporal resolution was used, while a daily time step was used to simulate snowmelt. For the other two modules, a daily time step was used for model calibration. The model performance was evaluated at the annual, seasonal, and daily time scales.

**Table 2:** Summary of the Scenarios

Scenario	Details
R	Estimation of all model parameters using only observed runoff data
R + Snowacc	Estimation of all model parameters using runoff and precipitation phase data
R + Snowmelt	Estimation of all model parameters except wet bulb temperature *T*_*wb*_ using runoff and snow cover data
R + ET	Estimation of soil moisture accounting and runoff generation parameters using runoff and actual evapotranspiration data
R + SM	Estimation of soil moisture accounting and runoff generation parameters using runoff and soil moisture data
R + 50ET + 50SM	Estimation of soil moisture accounting and runoff generation parameters using runoff, actual evapotranspiration (*w*_*ET*_ = 50%), and soil moisture (*w*_*SM*_ = 50%) data
R + ET + G	Estimation of runoff generation parameters using runoff and runoff generation data; the soil moisture accounting parameters were fixed in Scenario R + ET
R + SM + G	Estimation of runoff generation parameters using runoff and runoff generation data; the soil moisture accounting parameters were fixed in Scenario R + SM
R + 50ET + 50SM + G	Estimation of runoff generation parameters using runoff and runoff generation data; the soil moisture accounting parameters were fixed in Scenario R + 50ET + 50SM

**Table 3:** Scenarios (According to Table 2) and Corresponding, Calibrated Model Parameter Sets

Scenario name	Parameters													
	*SCF*	*DDF*	*T*_*wb*_	*T*_*m*_	*LPrat*	*FC*	*β*	*k*_*0*_	*k*_*1*_	*k*_*2*_	*LS*_*UZ*_	*c*_*P*_	*B*_*MAX*_	*c*_*R*_
R	**1.3**	**4.3**	**−0.1**	**0.0**	**0.0**	**319.4**	**0.6**	**1.1**	**4.7**	**84.7**	**9.2**	**1.5**	**5.3**	**20.0**
R + Snowacc	1.0	2.5	**0.6**	−0.9	0.1	109.4	1.3	1.0	5.2	97.5	9.0	1.6	7.5	40.1
R + Snowmelt	**0.9**	**3.1**	0.6	**−0.3**	0.0	118.5	0.9	1.0	4.2	100.1	8.6	1.5	2.3	32.3
R + ET	0.9	3.1	0.6	−0.3	**1.0**	**476.8**	**6.3**	1.0	6.8	123.2	10.4	0.9	4.8	21.3
R + SM	0.9	3.1	0.6	−0.3	**0.9**	**149.5**	**8.9**	0.3	3.9	142.2	1.2	3.6	12.3	40.2
R + 50ET + 50SM	0.9	3.1	0.6	−0.3	**1.0**	**177.8**	**19.6**	1.1	4.8	168.1	14.1	3.5	12.6	33.9
R + ET + G	0.9	3.1	0.6	−0.3	1.0	476.8	6.3	**0.1**	**26.6**	**156.4**	**2.0**	0.4	12.7	16.7
R + SM + G	0.9	3.1	0.6	−0.3	0.9	149.5	8.9	**0.2**	**17.1**	**169.4**	**1.0**	1.3	13.2	36.2
R + 50ET + 50SM + G	0.9	3.1	0.6	−0.3	1.0	177.8	19.6	**0.2**	**15.1**	**215.7**	**1.1**	1.4	12.2	37.5

*Note*. Parameters which were fixed at a certain scenario are shown in bold.

### 3.2 Model Calibration With Only Runoff Data

According to the first calibration approach (Scenario R), the model was calibrated to observed runoff by maximizing *Z_*1*_* (−) multiobjective function 16. The main idea was to capture the water balance by minimizing the relative volume error and to mimic the recession parts of the hydrographs. *Z*_*1*_ was maximized for calibrating all 14 model parameters, using the DEoptim R package for parameter optimization (Ardia, Boudt, et al., 2010; Ardia, Ospina Arango, et al., 2010; Ardia et al., 2016; Mullen et al., 2011).

(16) $$\begin{array}{c} Z_{1}=0.5\cdot {\text{lNash}}_{Q}+0.5\cdot {\mathrm{VE}}_{Q}\\ \text{where}\;{\mathrm{VE}}_{Q}=-{\mathrm{VE}}_{Q}\;\text{if}\;0<{\mathrm{VE}}_{Q} \end{array},$$

where *lNash*_*Q*_ (−) is the logarithmic Nash Sutcliffe efficiency for daily runoff according to 17

(17) $${\text{lNash}}_{Q}=1-\frac{\sum {\left(\text{logQ}-{\text{logQ}}_{o}\right)}^{2}}{\sum {\left({\text{logQ}}_{o}-\bar{\log\bar{Q_{o}}}\right)}^{2}},$$

where *Q* (mm/day) is daily simulated runoff, *Q*_*o*_ (mm/day) is daily observed runoff, and $\bar{Q_{o}}$ (mm/day) is the mean of the observed runoff.

*VE*_*Q*_ (−) (Criss & Winston, 2008) is the relative volume error for runoff according to 18

(18) $${\mathrm{VE}}_{Q}=\frac{\sum Q-\sum Q_{o}}{\sum Q_{o}}.$$

The modeling results were evaluated on three time scales: annual, seasonal, and daily. On the annual time scale, the volumes of observed and simulated runoff were compared, and the relative volume error was calculated according to 18. On the seasonal time scale, the monthly average observed and simulated daily runoff was compared. The monthly Kling‐Gupta efficiency *KGE*_*Q,m*_ (−) (Gupta et al., 2009) for runoff was calculated according to 19 using the hydroGOF R package (Zambrano‐Bigiarini, 2020)

(19) $$\begin{array}{c} {\mathrm{KGE}}_{Q,m}=1-{\mathrm{ED}}_{Q,m}\\ \;\text{where}\;{\mathrm{ED}}_{Q,m}=\sqrt{{\left(r_{Q,m}-1\right)}^{2}+{\left({\text{Beta}}_{Q,m}-1\right)}^{2}+{\left({\text{Alpha}}_{Q,m}-1\right)}^{2}} \end{array},$$

where *r*_*Q,m*_ (−) is the monthly Pearson correlation coefficient for runoff according to 20

(20) $$r_{Q,m}=\frac{\mathrm{cov}\left(Q_{m},Q_{m,o}\right)}{\sigma_{Q_{m}}\sigma_{Q_{m,o}}},$$

where *σ*_*Qm*_ (mm/day) and *σ*_*Qm,o*_ (mm/day) are the standard deviation of simulated and observed monthly average runoff, *Q*_*m*_ and *Q*_*m,o*_, respectively. *Beta*_*Q,m*_ (−) is the bias, that is, the ratio between the mean of the simulated monthly average runoff and the mean of the observed monthly average runoff. *Beta*_*Q,m*_ is ideally 1. *Alpha*_*Q,m*_ (−) is the variability ratio, that is, the ratio between the standard deviation of the simulated monthly average runoff and the standard deviation of the observed monthly average runoff. *Alpha*_*Q,m*_ is ideally 1.

On the daily time scale, daily logarithmic Nash Sutcliffe efficiency for runoff *lNash*_*Q*_ was calculated according to 17.

### 3.3 Model Calibration With Runoff and Additional Data

We divided the model parameter estimation procedure into separate steps, looking at the processes associated with the three modules of the model (snow, soil moisture, and runoff generation), which were linked to field observations. Using runoff and additional data, the 14 free parameters were gradually fixed, step‐by‐step, proceeding along the three modules of the model. This sequence was chosen to follow the main direction of the movement of water in the water cycle. First, all 14 parameters were calibrated using runoff and snow data. In the next step, the snow parameters were fixed, and only the soil moisture and runoff generation parameters were calibrated using runoff and actual evapotranspiration and/or soil moisture data. In the last step, the snow and soil moisture parameters were fixed, and only the runoff generation parameters were calibrated using runoff, overland flow, and storage change data.

#### 3.3.1 Snow Parameters

**Figure 4:**
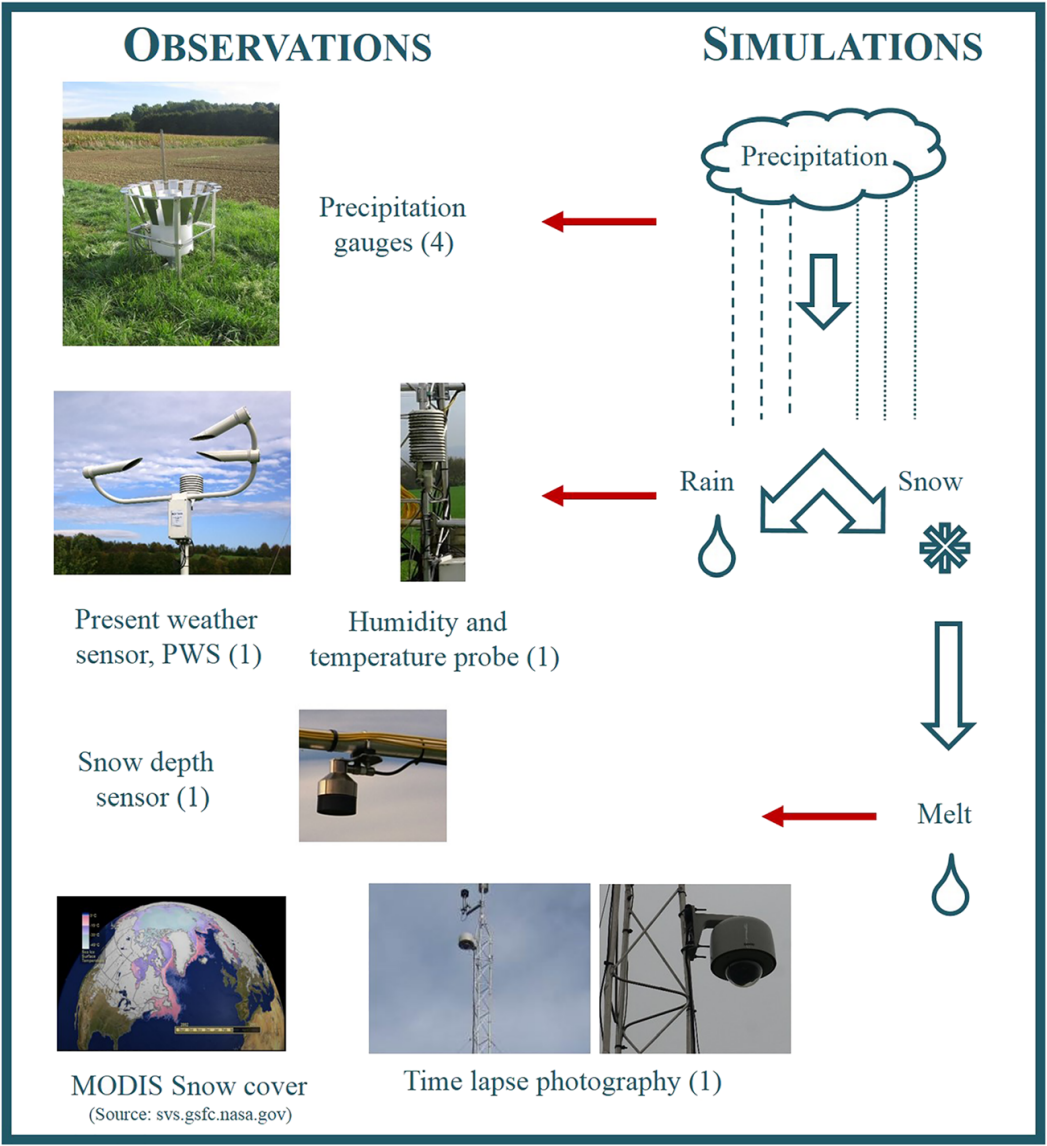
Linking observations with Hydrologic Model Simulations I: snow module (number of sensors is indicated in brackets).

Simulation of the snow accumulation, that is, the phase of precipitation (Scenario R + Snowacc), and snowmelt (Scenario R + Snowmelt) was optimized using observations of runoff, four precipitation gauges, the present weather sensor, time‐lapse photos of snow cover in the catchment, MODIS snow cover images, and data of the snow depth sensor (Figure 4).

Depending on the size and velocity of the precipitation measured by the present weather sensor, the observed precipitation *P*, which was calculated as the average of the four gauges, was assigned an output code describing its type (Figure 4). The output codes (PWS100 SYNOP 4680 codes) with 1 min temporal resolution were resampled to half‐hourly resolution, using the median, to match the half‐hourly temperature measurements. The resampled output codes were assigned to one of the four categories: no data (0), no precipitation (1), rain (2), and snow (3).

All 14 free parameters were optimized to fit the modeled runoff and phase of the precipitation to the observed one. *Z*_*2*_ (−) multiobjective function was maximized using the DEoptim R package for parameter optimization, according to 21

(21) $$\begin{array}{c} Z_{2}=0.1\cdot {\text{lNash}}_{Q}+0.1\cdot {\mathrm{VE}}_{Q}-10\cdot Z_{\text{Snowacc}}\\ \text{where}\;{\mathrm{VE}}_{Q}=-{\mathrm{VE}}_{Q}\;\text{if}\;0<{\mathrm{VE}}_{Q} \end{array},$$

where *Z*_*Snowacc*_ (−) is the number of half hours with poor phase simulations according to 22

(22) $$Z_{\text{Snowacc}}=n_{\text{false}}$$

where *n*_*false*_ (−) is the number of those half hours, when the model simulated precipitation phase (rain, snow, or no precipitation) did not agree with the observed precipitation phase. The weights on the single objectives were found with sensitivity analysis.

Out of the optimum parameter set, the wet bulb temperature parameter *T*_*wb*_ was kept constant in the following optimization steps.

In order to optimize the snowmelt simulations, an observed snow cover index *SCI*_*o*_ was set up, showing if snow was observed (1) or not (0) in the catchment on a daily basis, based on three types of observations (Figure 4):

1. Time‐lapse photos were manually checked: If snow cover was visible in the catchment, *SCI*_*o*_ was assigned 1 (Snow), otherwise 0 (No Snow).
2. During periods when time‐lapse photos were not available due to camera malfunction or power outage, daily MODIS Normalized Difference Snow Index (NDSI) images were checked (Hall & Riggs, 2016a, 2016b). The territory of the HOAL was extracted and reprojected (MRT, 2004) from the h19v06 MODIS tile with 500 m spatial resolution. Out of the eight pixels, five cover more than 50% of the catchment area; therefore, the average of these five pixels was calculated. The catchment average MODIS NDSI values were reclassified to three categories: snow (40 ≤ NDSI ≤ 100), no snow (0 ≤ NDSI < 40), and no data (100 < NDSI), choosing 40 as a threshold based on Dozier (1989). If the NDSI values were classified to category snow or no snow, the composite snow cover index was assigned 1 or 0, respectively.
3. If the NDSI values were classified as no data, the snow sensor data were checked. If the recorded snow depth was above 0 cm, the composite snow cover index *SCI*_*o*_ was assigned 1, otherwise 0.

The simulated snow cover index *SCI* was assigned 1 (snow observed in the catchment), if the modeled snow water equivalent *SWE* (mm) was larger than 2 mm. Otherwise, it was assigned 0. The 2 mm threshold was chosen based on sensitivity analyses.

The 13 free parameters (all parameters except *T*_*wb*_ wet bulb temperature, which was fixed in the previous step) were optimized to fit the modeled runoff and snow cover index *SCI* to the observed one. *Z*_*3*_ (−) multiobjective function was maximized using the DEoptim R package for parameter optimization, according to 23

(23) $$\begin{array}{c} Z_{3}=0.1\cdot {\text{lNash}}_{Q}+0.1\cdot {\mathrm{VE}}_{Q}-10\cdot Z_{\text{Snowmelt}}\\ \text{where}\;{\mathrm{VE}}_{Q}=-{\mathrm{VE}}_{Q}\;\text{if}\;0<{\mathrm{VE}}_{Q} \end{array},$$

where *Z*_*Snowmelt*_ (−) is the number of days with poor snow cover index simulations similarly to 22, that is, the simulated snow cover index did not agree with the observed one.

Out of the optimum parameter set, the remaining three snow parameters (*SCF*, *DDF*, and *T*_*m*_) were kept constant in the following optimization steps.

The modeling efficiency in terms of simulating snow accumulation and snowmelt was evaluated by analyzing *Z*_*Snowacc*_ and *Z*_*Snowmelt*_ for the scenario, when only runoff was used for model calibration (Scenario R) and for the scenarios when additional information on snow was used besides runoff (Scenarios R + Snowacc and R + Snowmelt, respectively).

#### 3.3.2 Soil Moisture and Evapotranspiration Parameters

**Figure 5:**
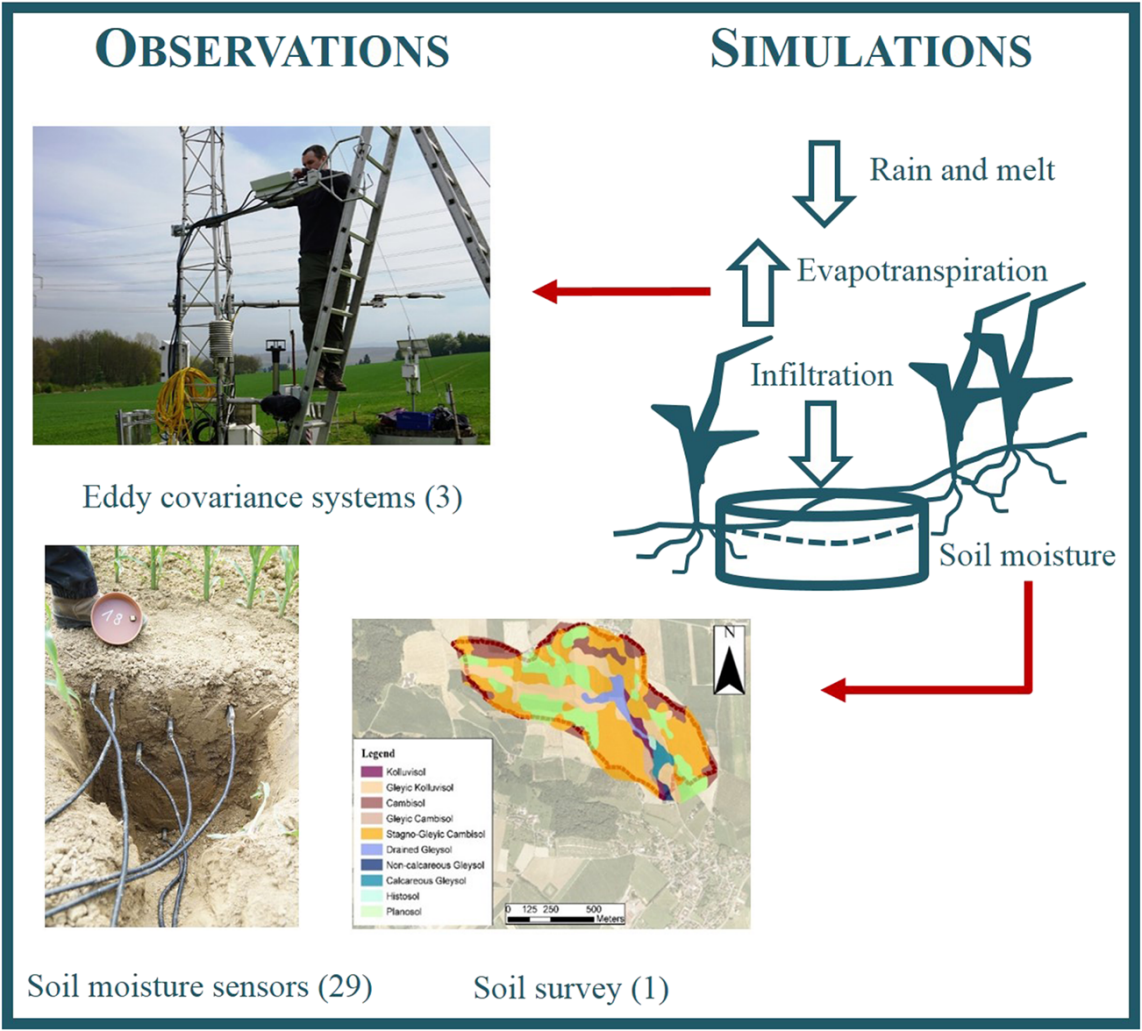
Linking observations with Hydrologic Model Simulations II: soil moisture accounting module (number of sensors is indicated in brackets).

Simulation of catchment average soil moisture and evapotranspiration was optimized using runoff data and observations of the soil moisture sensors, the eddy covariance systems, and results from a soil survey (Figure 5).

For actual evapotranspiration, a catchment average evapotranspiration was calculated for each day. Measurements of the closed path eddy at the weather station (measuring grass evapotranspiration) and the two mobile eddies (measuring evapotranspiration from different crop types, such as maize, winter wheat, winter barley, or bare soil) were weighted with the area of the different land use types. For the riparian forest close to the stream, due to the lack of evapotranspiration measurements, a crop coefficient was used. The grass evapotranspiration from the weather station eddy was then multiplied with this crop coefficient. A similar method was used in case of data gaps or in case the evapotranspiration rates from a certain main crop type were not measured in a certain year.

For soil moisture, the average of all stations over all depths was considered. To make the simulated soil moisture comparable with the measurements, the soil moisture time series were standardized according to 24

(24) $$\mathrm{SMs}=\frac{\mathrm{SM}-\bar{\mathrm{SM}}}{\sigma_{\mathrm{SM}}},$$

where *SMs* (−) is the simulated standardized soil moisture, *SM* (mm) is the simulated soil moisture, and $\bar{\mathrm{SM}}$ (mm) and *σ*_*SM*_ (mm) are the average and the standard deviation of the simulated soil moisture over each period (calibration and validation periods separately). The observed standardized soil moisture *SMs*_*o*_ (−) was calculated similarly to 24.

A multiobjective function *Z*_*4*_ (−) 25 was maximized for calibrating 10 model parameters, using the DEoptim R package. The snow module parameters (optimized according to section 3.3.1) were not changed in this step. *Z*_*4*_ combined runoff efficiency, the daily Nash Sutcliffe efficiency for evapotranspiration *Z*_*ET*_, and standardized soil moisture *Z*_*SM*_ with different weights according to 25

(25) $$\begin{array}{c} Z_{4}=0.25\cdot {\text{lNash}}_{Q}+0.25\cdot {\mathrm{VE}}_{Q}+0.5\cdot \left(w_{\mathrm{ET}}{\text{Nash}}_{\mathrm{ET}}+w_{\mathrm{SM}}{\text{Nash}}_{\mathrm{SMs}}\right)\\ \text{where}\;{\mathrm{VE}}_{Q}=-{\mathrm{VE}}_{Q}\;\text{if}\;0<{\mathrm{VE}}_{Q} \end{array},$$

where *w*_*ET*_ (−) is the weight on the evapotranspiration objective, ranging between 0 and 1, and *w*_*SM*_ (−) is the weight on the soil moisture objective according to 26

(26) $$w_{\mathrm{SM}}=1-w_{\mathrm{ET}}$$

*Nash*_*ET*_ is the daily Nash Sutcliffe efficiency for actual evapotranspiration 27

(27) $${\text{Nash}}_{\mathrm{ET}}=1-\frac{\sum {\left(\mathrm{ET}-{\mathrm{ET}}_{o}\right)}^{2}}{\sum {\left({\mathrm{ET}}_{o}-\bar{{\mathrm{ET}}_{o}}\right)}^{2}},$$

where *ET* (mm/day) is the simulated actual evapotranspiration, *ET*_*o*_ (mm/day) is observed actual evapotranspiration, and $\bar{{\mathrm{ET}}_{o}}$ (mm/day) is the mean of the observed actual evapotranspiration. The Nash Sutcliffe efficiency for the daily standardized soil moisture *Nash*_*SMs*_ (−) was calculated similarly to 27 with $\bar{{\mathrm{SMs}}_{o}}=0$.

Results of a soil survey performed on a 50 × 50 m raster were used to constrain the field capacity *FC*. The upper boundary of *FC* was set to 450 mm considering that the maximum of the observed, depth‐averaged field capacity was 430 mm in the catchment (Murer et al., 2004).

This optimization step was repeated 10 times, to check the stability of the optimized model parameters. Out of the optimum parameter set, the three soil moisture parameters (*LP*_*rat*_, *FC*, and *β*) were kept constant in the following optimization steps. Three main scenarios were chosen for further analysis. In Scenario R + ET, *w*_*ET*_ was chosen as 1; therefore, only evapotranspiration information was used besides runoff in the multiobjective function *Z*_*4*_. In Scenario R + SM, *w*_*ET*_ was chosen as 0; therefore, only soil moisture information was used besides runoff in the multiobjective function *Z*_*4*_. In Scenario R + 50ET + 50SM, *w*_*ET*_ was chosen as 0.5; therefore, both evapotranspiration and soil moisture information were used besides runoff in the multiobjective function *Z*_*4*_. These parameters were not changed in the following optimization step.

The modeling efficiency in terms of simulating soil moisture and actual evapotranspiration was evaluated by analyzing the relative volume error for actual evapotranspiration *VE*_*ET*_ similarly to 18 and the monthly Kling‐Gupta efficiency according to 19 for actual evapotranspiration and standardized soil moisture, *KGE*_*ET,m*_ and *KGE*_*SMs,m*_, respectively, as a function of weight on the evapotranspiration objective *w*_*ET*_. Furthermore, the annual (relative volume error for actual evapotranspiration) and monthly performances (monthly average actual evapotranspiration rates and standardized soil moisture) were also compared for selected scenarios (Scenarios R, R + ET, R + SM, and R + 50ET + 50SM).

#### 3.3.3 Runoff Generation Parameters

**Figure 6:**
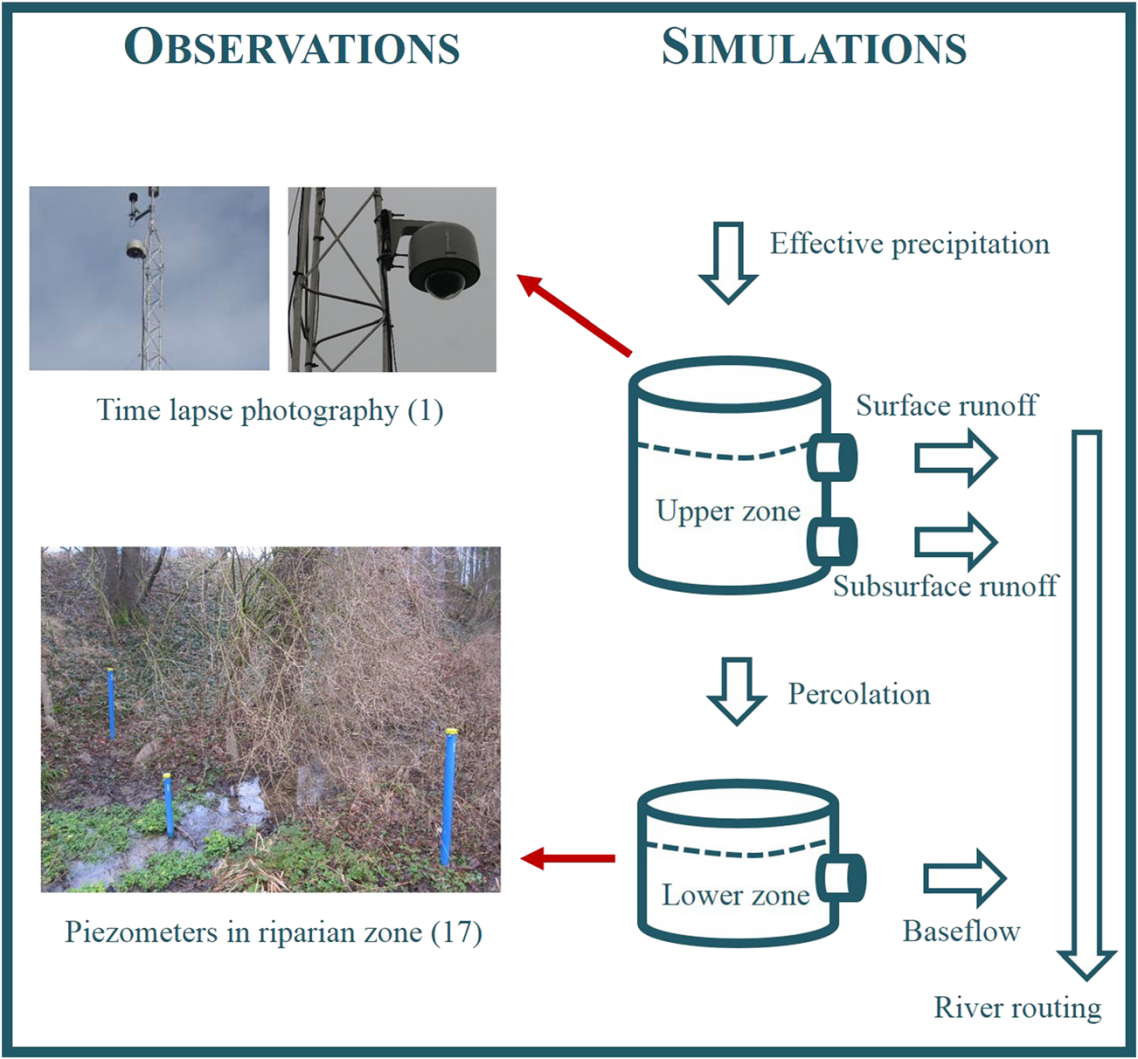
Linking observations with Hydrologic Model Simulations III: runoff generation module (number of sensors is indicated in brackets).

The model parameters related to the subsurface dynamics were optimized using runoff data, time‐lapse photos of saturation patterns, and piezometer measurements of groundwater levels (Figure 6).

In order to optimize the very fast runoff *q*_*0*_ simulations, saturation excess runoff events were identified in the valley bottom located in the centroid of the catchment by a digital camera at the weather station according to Silasari et al. (2017). The days when the model simulated very fast runoff (*q*_*0*_) were calibrated to the dates when overland flow events were observed by the camera.

Monthly average storage change in the lower zone reservoir d*S*_*LZ*_ (mm/month) was optimized using piezometer observations of groundwater levels *GWL*, located in the riparian zone. Generally, groundwater levels are higher in the riparian zone close to the stream compared to the catchment average groundwater levels. This might introduce bias and potentially different temporal dynamics. However, based on more recent data, that is, piezometric data from deep boreholes, which are located not only in the riparian zone but also on the slopes and ridgelines of the catchment, this bias was likely small. The dynamics of the deeper riparian piezometers located on the right side of the stream matched the dynamics of the deep borehole piezometers. Riparian groundwater level data with 5 min temporal resolution were averaged to daily values, and missing data were spline interpolated for each riparian piezometer. The daily, gap‐filled time series were aggregated to monthly values. The monthly average groundwater level data were differentiated in time, and the difference was multiplied by drainable porosity 0.036 to get the monthly storage change values d*S*_*o*_ (mm/month). The storage change calculated for each piezometer was then spatially interpolated using Thiessen polygons, and a catchment average storage change was calculated from the interpolated raster map. The simulated monthly average standardized lower zone storage change d*Ss* (−) was calibrated to the observed catchment average standardized storage change d*Ss*_*o*_ (−). The observed and simulated standardized storage change time series were calculated analogously to 24.

A multiobjective function *Z*_*5*_ (−) 28 was maximized for calibrating seven model parameters, using the DEoptim R package. The snow module and soil moisture accounting module parameters (optimized according to sections 3.3.1 and 3.3.2) were not changed in this step. *Z*_*5*_ combined runoff efficiency, the relative number of days with correctly simulated very fast runoff *Z*_*OF*_ (−), and the relative number of months with correctly simulated sign of the standardized storage change *Z*_*dSs*_ (−) with different weights

(28) $$\begin{array}{c} Z_{5}=0.25\cdot {\text{lNash}}_{Q}+0.25\cdot {\mathrm{VE}}_{Q}+0.5\cdot \left(w_{\text{OF}}Z_{\text{OF}}+w_{\mathrm{dS}}Z_{\mathrm{dSs}}\right)\\ \text{where}\;{\mathrm{VE}}_{Q}=-{\mathrm{VE}}_{Q}\;\text{if}\;0<{\mathrm{VE}}_{Q} \end{array},$$

where *w*_*OF*_ (−) is the weight on the overland flow *OF* objective, ranging between 0 and 1, and *w*_*dSs*_ (−) is the weight on the storage change objective according to 29

(29) $$w_{\mathrm{dSs}}=1-w_{\text{OF}}$$

*Z*_*OF*_ (−) is the relative number of days, when very fast runoff *q*_*0*_ was correctly simulated 30

(30) $$Z_{\text{OF}}=\frac{n_{q0}}{n_{\text{OF}}}$$

where *n*_*q0*_ (−) is the number of those days when the model simulated very fast runoff *q*_0_ and overland flow was simultaneously observed by time‐lapse photos taken by the camera located at the weather station and *n*_*OF*_ (−) is the total number of days, when overland flow was observed in the catchment.

*Z*_*dSs*_ (−) is the relative number of months, when the model correctly simulated the sign of the standardized storage change 31

(31) $$Z_{\mathrm{dSs}}=\frac{n_{\mathrm{sgn}\left(\mathrm{dSs}\right)}}{n_{\mathrm{dSs},o}}$$

where *n*_*sgn*(*dSs*)_ (−) is the number of those months when the sign of the model simulated standardized lower zone storage change was the same as the sign of the observed standardized storage change and *n*_*dSs,o*_ (−) is the total number of months when storage change was observed.

This optimization step was repeated 10 times to check the stability of the optimized model parameters. Three main scenarios were chosen for detailed analysis: R + ET + G, R + SM + G, and R + 50ET + 50SM + G. In each scenario, equal weights were put on the overland flow (*w*_*OF*_ = 0.5) and storage change (*w*_*dS*_ = 0.5) objectives.

The modeling efficiency in terms of simulating overland flow was evaluated by analyzing *Z*_*OF*_ as a function of *w*_*OF*_. The modeling efficiency in terms of simulating storage change in the saturated zone was assessed by *Z*_*dS*s_ as a function of *w*_*OF*_.

#### 3.3.4 Runoff Simulation

The modeling efficiency in terms of simulating runoff was evaluated on annual, monthly, and daily time scales. On the annual time scale, the volumes of observed and simulated runoff were compared. On the seasonal time scale, monthly average observed and simulated runoff time series were compared. On the daily time scale, daily runoff time series were compared.

## 4 Results

### 4.1 Model Calibration With Only Runoff Data

When the model was calibrated using only runoff data, the performance of runoff simulations was very good during the calibration period on each time scale (Table 4). During model validation, the annual and seasonal performances of runoff simulations were still good. The relative volume error was below 20% and the monthly Pearson correlation coefficient was around 0.75, the monthly bias *Beta*_*Q,m*_ was close to 1.0, and the monthly variability ratio *Alpha*_*Q,m*_ was also close to 1.0 during the long validation period, and it was around 0.7 during the shorter, 2 year long validation period. But the daily performance was very low, the daily logarithmic Nash Sutcliffe efficiency for runoff was around 0.2 during both periods.

**Table 4:** Model Calibration Using Runoff Data Alone or With Additional Data: Performance of Runoff *Q* (Relative Volume Error *VE*_*Q*_, Three Components of Monthly Kling‐Gupta Efficiency Such as *r*_*Q,m*_, *Beta*_*Q,m*_, and *Alpha*_*Q,m*_, and Daily Logarithmic Nash Sutcliffe Efficiency *lNash*_*Q*_) During Model Calibration (2013–2015) and First and Second Validation (1991–2012 and 2016–2017, Respectively) Periods

Scenario	Calibration period (2013–2015)			Validation period 1 (1991–2012)			Validation period 2 (2016–2017)		
	*VE*_*Q*_ (−)	*r*_*m,Q*_ (−) *Beta*_*m,Q*_ (−) *Alpha*_*m,Q*_ (−)	*lNash*_*Q*_ (−)	*VE*_*Q*_ (−)	*r*_*m,Q*_ (−) *Beta*_*m,Q*_ (−) *Alpha*_*m,Q*_ (−)	*lNash*_*Q*_ (−)	*VE*_*Q*_ (−)	*r*_*m,Q*_ (−) *Beta*_*m,Q*_ (−) *Alpha*_*m,Q*_ (−)	*lNash*_*Q*_ (−)
R	0.00	0.98	0.81	0.18	0.74	0.22	0.15	0.75	0.17
		1.00			1.16			1.15	
		1.04			1.18			0.71	
R + Snowacc	0.00	0.94	0.81	0.28	0.74	0.18	0.15	0.85	0.27
		1.00			1.26			1.14	
		1.01			1.60			0.90	
R + Snowmelt	0.00	0.96	0.82	0.28	0.74	0.19	0.17	0.87	0.27
		1.00			1.26			1.17	
		0.97			1.54			0.87	
R + ET	0.00	0.96	0.67	0.18	0.76	0.16	0.03	0.62	−0.52
		1.00			1.16			1.02	
		1.05			1.49			0.98	
R + SM	0.00	0.95	0.72	0.21	0.74	0.15	0.05	0.83	0.25
		1.00			1.20			1.05	
		1.09			1.52			0.93	
R + 50ET + 50SM	0.01	0.95	0.70	0.21	0.74	0.17	0.01	0.85	0.20
		1.01			1.19			1.00	
		1.15			1.49			0.86	
R + ET + G	−0.01	0.85	0.52	0.18	0.70	0.05	0.02	0.57	−0.62
		0.99			1.16			1.01	
		0.88			1.36			1.10	
R + SM + G	0.00	0.91	0.63	0.21	0.75	0.10	0.03	0.81	0.26
		1.00			1.20			1.03	
		0.99			1.44			0.89	
R + 50ET + 50SM + G	0.00	0.92	0.59	0.20	0.76	0.09	−0.01	0.82	0.18
		1.00			1.19			0.99	
		0.97			1.38			0.87	

*Note*. Scenarios according to Table 2.

### 4.2 Model Calibration With Runoff and Additional Data

#### 4.2.1 Snow Simulation

The first step of the step‐by‐step model parameter estimation was to find the optimal snow parameters.

Calibrating the model parameters to runoff and the observations from the present weather sensor (Scenario R + Snowacc) gave 0.31% and 0.40% of poor simulation times steps, that is, the phase of the model simulated precipitation differed from the observed one, in the calibration and validation periods, respectively. This was slightly better than the simulations that used only runoff data for calibration instead (Scenario R) (Table 5). Compared to Scenario R, in Scenario R + Snowacc, the calibrated wet bulb temperature *T*_*wb*_ increased from −0.1°C to 0.6°C (Table 3). This value is more realistic considering that it is closer to 1.0°C, which is the median of the wet bulb temperature observed in the catchment during precipitation events with a shift in precipitation phase (calculated according to Text S2).

**Table 5:** Performance of Snow Accumulation and Snowmelt Simulations: Number of Time Steps With Poor Snow Accumulation Simulations, That Is, Those Time Steps When the Model Simulated Precipitation Phase Did Not Agree With the Observed One (Numbers in Brackets Indicating Those Relative Number of Time Steps When the Phase of Precipitation Was Snow), and Snow Cover Simulations, That Is, Those Time Steps When the Model Simulated Snow Cover Index Did Not Agree With the Observed One, Relative to the Number of Time Steps With Observations

Scenario	Relative number of time steps with poor snow accumulation simulations (snow phase) (%)		Scenario	Relative number of time steps with poor snowmelt simulations (%)	
	Calibration period (2013–2015)	Validation period 2 (2016–2017)		Calibration period (2013–2015)	Validation period 2 (2016–2017)
R	0.45 (0.34)	0.52 (0.48)	R	4.66	7.25
R + Snowacc	0.31 (0.11)	0.40 (0.18)	R + Snowmelt	4.38	6.29
Number of half hourly time steps with observations	35,626	23,972	Number of daily time steps with observations	1,095	731

*Note*. Scenarios according to Table 2: Scenario R where only runoff was used for model calibration and Scenarios R + Snowacc and R + Snowmelt where runoff and snow data were used for model calibration.

Calibrating the snowmelt parameters to the observed snow cover index *SCI*_*o*_ (Scenario R + Snowmelt) gave 4.4% and 6.3% of poor simulation time steps which, again, was better than Scenario R. In Scenario R, the calibrated snow correction factor *SCF* indicated 30% increase in snowfall precipitation, which was higher than expected for lowland catchments (Table 3). The lower *SCF* values in Scenarios R + Snowacc and R + Snowmelt were much more realistic (Table 3). The higher snow correction factor *SCF* in Scenario R was then compensated by more intense snowmelt, that is, higher degree day factor *DDF* model parameter, which was much higher than expected and found in flatland catchments in Austria (Merz et al., 2011; Sleziak et al., 2020).

The trade‐off between simulating runoff and snowmelt accurately was not large, that is, using additional information on snow did not have a large influence on runoff simulations during the calibration period (Table 4), but it changed the simulation results during the 22 year long validation period by causing larger relative volume error, higher monthly variability ratio, and lower logarithmic Nash Sutcliffe efficiency for runoff. During the second, 2 year long validation period, the runoff simulation performance on the seasonal and daily time scales generally improved (Table 4).

#### 4.2.2 Soil Moisture and Evapotranspiration Simulation

The second step of the step‐by‐step parameter estimation was to fix the parameters of the soil moisture accounting module.

**Figure 7:**
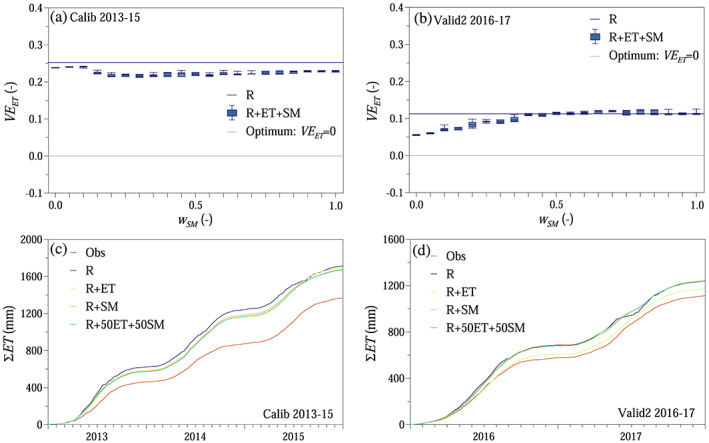
Soil moisture and evapotranspiration simulations: (a and b) relative volume error for evapotranspiration *VE*_*ET*_ as a function of weight on soil moisture *w*_*SM*_ in the compound objective function shown as boxplots (R + ET + SM) and Scenario R, when only runoff was used for calibration. (c and d) Simulated cumulative actual evapotranspiration *ET* when the model was calibrated only with runoff and evapotranspiration (R + ET), only with runoff and standardized soil moisture (R + SM), and a combination of *w*_*ET*_ = 0.5 evapotranspiration and *w*_*SM*_ = 0.5 standardized soil moisture (R + ET + SM). For comparison, observations (Obs) and Scenario R are shown. Scenarios according to Table 2.

The smallest relative volume errors of evapotranspiration were achieved for soil moisture weights of *w*_*SM*_ = 0.3 and *w*_*SM*_ = 0.0 in the calibration and validation periods based on the median of 10 model runs (Figures 7a and 7b). Generally, the model tended to overestimate evapotranspiration (Figures 7c and 7d). This could be a consequence of using the Nash Sutcliffe efficiency for evapotranspiration in the objective function, where the model was fitted to the peaks and not lower values of evapotranspiration rates. Furthermore, there is a mismatch between field observations and the HBV type, soil moisture‐dependent evapotranspiration calculations. For example, during precipitation events, measured evapotranspiration drops to zero, while model simulations increase due to the higher soil moisture content. During model validation, the relative volume error for evapotranspiration was closer to zero, especially when using only evapotranspiration information in the objective function (Figures 7b and 7d).

**Figure 8:**
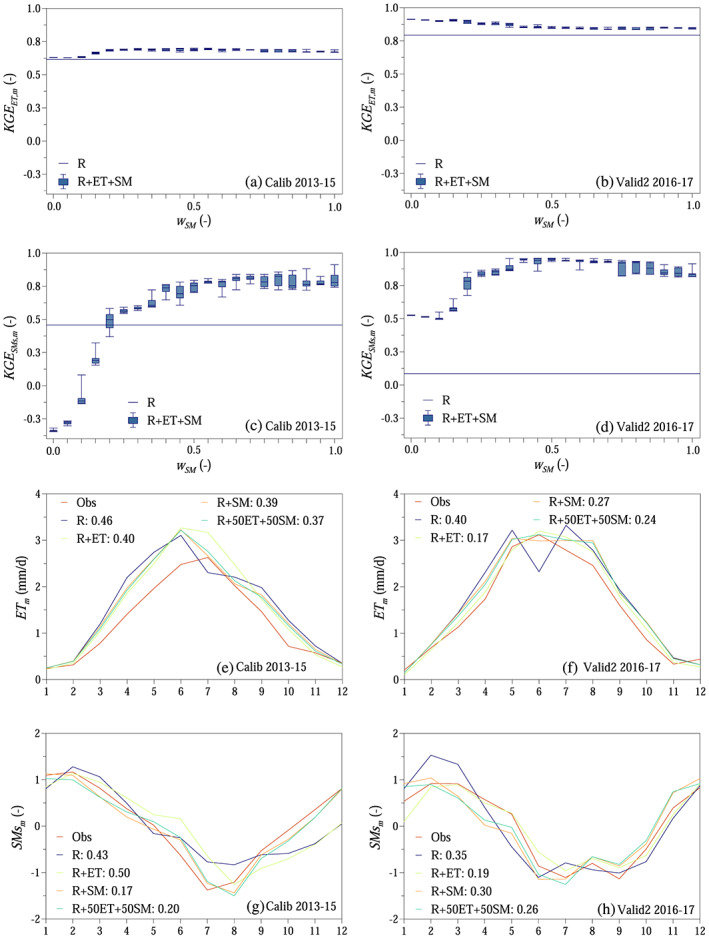
Soil moisture and evapotranspiration simulations: (a–d) monthly Kling‐Gupta efficiencies for evapotranspiration (*ET*) and standardized soil moisture (*SMs*) as a function of weight on soil moisture *w*_*SM*_ in the compound objective function shown as boxplots (R + ET + SM) and R scenario. (e–h) Monthly averages for *ET* and *SMs* for different scenarios as in Figure 7, according to Table 2. Numbers in legend indicate root mean squared error.

The monthly Kling‐Gupta efficiency for evapotranspiration was above 0.6 both during model calibration and validation (Figures 8a and 8b). The monthly Kling‐Gupta efficiency for standardized soil moisture was slightly lower, if only evapotranspiration was used in the objective function (Figures 8c and 8d). When both evapotranspiration and soil moisture objectives were involved in the objective function, the proposed approach outperformed Scenario R (Figures 8a–8d). The root mean squared error between simulated and observed monthly average evapotranspiration and standardized soil moisture was generally lower, when besides runoff extra information on evapotranspiration and/or soil moisture were involved in the model calibration (Figures 8e–8h).

For further analysis, calibrated model parameters from three main scenarios were chosen, when a weight of *w*_*SM*_ = 0.0 (R + ET), *w*_*SM*_ = 1.0 (R + SM), and *w*_*SM*_ = 0.5 (R + 50ET + 50SM) was used on the soil moisture objective in the objective function when calibrating the soil moisture accounting module.

In Scenario R, the parameter related to the limit for potential evapotranspiration *LPrat* was very close to zero, meaning that actual evapotranspiration was almost always reaching its potential rate, even when the soil was very dry (Table 3). If evapotranspiration and soil moisture were used for model calibration, *LPrat* was closer to 1.0. This means that a certain wetness in the soils, that is, more water, was needed to reach potential evapotranspiration (Table 3). The nonlinear parameter for runoff production *β* was below 1.0, if only runoff was used for model calibration (Scenario R; Table 3), meaning that more water was allocated for runoff and less for soil moisture storage. Considering the clayish soil types in the HOAL, this was highly unrealistic. *β* was well above 1.0, if actual evapotranspiration and/or soil moisture were also used for model calibration (Scenarios R + ET, R + 50ET + 50SM, and R + SM). This refers to a more nonlinear runoff generation, which would be expected in the catchment considering the soil types. According to the soil survey, the catchment average field capacity *FC* was around 400 mm. This means that the calibrated field capacities for Scenarios R + ET and R seemed more realistic. However, when checking the simulated storage time series, the Scenarios R + 50ET + 50SM and R + SM stood much closer to reality. In Scenario R, when only runoff was used for model calibration, the root zone soil storage remained well below saturation, and it even reached 0 mm in the summer months. This was not realistic, and it was not supported by the soil moisture observations in the catchment. When soil moisture and actual evapotranspiration data were used together for model calibration, the root zone soil storage never dried out. In Scenario R + ET, most of the water was stored in the soil, the upper zone storage was only a few mm during events, and the lower zone storage was around four times smaller. When also soil moisture was used for model calibration, the water was more evenly distributed between the storage elements (Scenarios R + SM and R + 50ET + 50SM).

The results indicate a clear trade‐off between accurately simulating actual evapotranspiration, soil moisture, and runoff. Using additional information unequivocally improved evapotranspiration and soil moisture simulations, especially on the daily time scale (Table 6). Using additional information on evapotranspiration and soil moisture besides runoff generally improved runoff simulations during the 2 year long validation period, while it only slightly deteriorated the results during model calibration and the 22 year long validation periods (Table 4). The difference between the scenarios in terms of runoff efficiency was generally small during model calibration and the 22 year long validation period. Compared to the 22 year long validation, the volume error reduced by order of magnitude, and the daily runoff simulation performance improved for the 2 year validation (Table 4). While soil moisture information improved runoff efficiency, evapotranspiration information deteriorated it during the 2 year long validation period (Table 4).

**Table 6:** Model Calibration Using Runoff, Soil Moisture, and Evapotranspiration Data: Performance of Evapotranspiration *ET* and Standardized Soil Moisture *SMs* Simulations (Relative Volume Error *VE*, Three Components of Monthly Kling‐Gupta Efficiency Such as *r*_*m*_, *Beta*_*m*_, and *Alpha*_*m*_, and Daily Nash Sutcliffe Efficiency *Nash*) During Model Calibration (2013–2015) and Second Validation (2016–2017) Periods

Variable	Scenario	Calibration period (2013–2015)			Validation period 2 (2016–2017)		
		*VE* (−)	*r*_*m*_ (−) *Beta*_*m*_ (−) *Alpha*_*m*_ (−)	*Nash* (−)	*VE* (−)	*r*_*m*_ (−) *Beta*_*m*_ (−) *Alpha*_*m*_ (−)	*Nash* (−)
*ET*	R	0.25	0.82	−0.54	0.11	0.88	0.15
			1.25			1.11	
			1.22			1.13	
	R + ET	0.24	0.96	0.52	0.05	0.99	0.78
			1.24			1.05	
			1.28			1.07	
	R + SM	0.23	0.88	0.39	0.12	0.95	0.68
			1.23			1.12	
			1.19			1.10	
	R + 50ET + 50SM	0.22	0.91	0.45	0.11	0.97	0.72
			1.22			1.11	
			1.20			1.10	
*SMs*	R		0.87	0.71		0.81	0.60
			0.47			1.89	
			0.98			1.02	
	R + ET		0.84	0.70		0.83	0.64
			−0.33			1.44	
			1.02			1.03	
	R + SM		0.99	0.96		0.94	0.86
			0.83			1.06	
			1.00			1.00	
	R + 50ET + 50SM		0.99	0.96		0.95	0.88
			0.75			0.98	
			1.00			1.01	

*Note*. Scenarios according to Table 2.

#### 4.2.3 Runoff Generation and Routing Simulation

The third step of the step‐by‐step parameter estimation was to fix the parameters of the runoff generation and routing module.

**Figure 9:**
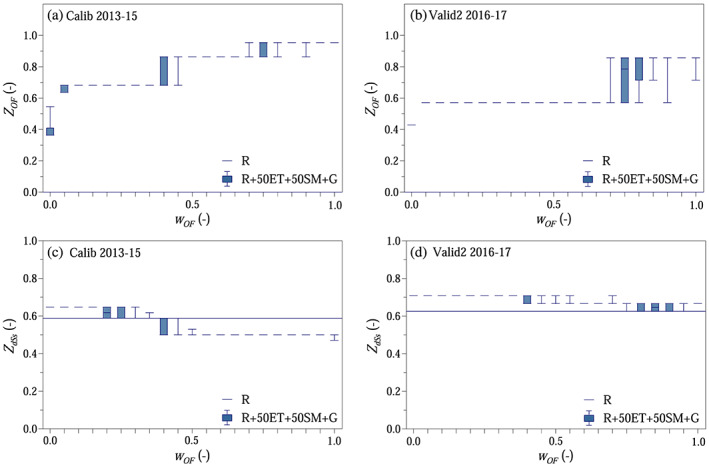
Overland flow and storage change simulations: (a and b) relative number of days with good overland flow simulations *Z*_*OF*_ as a function of weight on overland flow part *w*_*OF*_ in the compound objective function (containing overland flow and storage change objectives) shown as boxplots. Scenario R shown as line. (c and d) Relative number of months when the model correctly simulated the sign of the standardized storage change *Z*_*dSs*_ as a function of weight on overland flow part *w*_*OF*_ in the compound objective function shown as boxplots. Scenario R shown as line.

Using the parameters from Scenario R + 50ET + 50SM from the previous step, the model was run 10 times (Figure 9). The median of the relative number of days with good overland flow simulations, that is, days when the model simulated very fast runoff (*q*_*0*_) and overland flow were simultaneously observed, immediately exceeded 0.5 as soon as the weight on the overland flow part in the compound objective function was larger than zero (Figures 9a and 9b). In terms of overland flow simulations, the results outperformed Scenario R for each *w*_*OF*_ weight, as the model did not simulate overland flow at all when only runoff was used for model calibration. The median of the relative number of months with good storage change simulations, that is, months when the sign of the model simulated storage change agreed with the sign of the observed storage change, became gradually 10–20% lower as the weight on the overland flow objective increased, and during calibration, the results also underperformed Scenario R (Figures 9c and 9d).

Compared to Scenario R, when runoff generation data were also used for model calibration, the calibrated very fast storage time *k*_*0*_ became smaller (1.1 day for Scenario R and below 0.5 day if runoff generation data were also used for model calibration; Table 3), while the fast and slow storage times, *k*_*1*_ and *k*_*2*_, respectively, increased (Table 3). Faster overland flow and slower subsurface runoff correspond well with field observations. Overland flow events usually last a few hours according to camera observations, while the outflow from the subsurface reservoirs take several months due to the heterogeneous subsurface properties of the catchment.

For further analysis, the runoff generation parameters of the three main scenarios (R + ET + G, R + SM + G, and R + 50ET + 50SM + G) were calibrated by choosing the weight on the overland flow objective as *w*_*OF*_ = 0.5.

Regarding the trade‐off between simulating runoff and runoff generation processes accurately, by using additional information on overland flow and storage change besides runoff further improved runoff simulations during the 2 year long validation period, while it further deteriorated the daily performance during model calibration and the 22 year long validation (Table 4). Generally, the scenarios when either only soil moisture or both soil moisture and evapotranspiration were used in the model calibration performed the best (Table 4).

#### 4.2.4 Runoff Simulation

**Figure 10:**
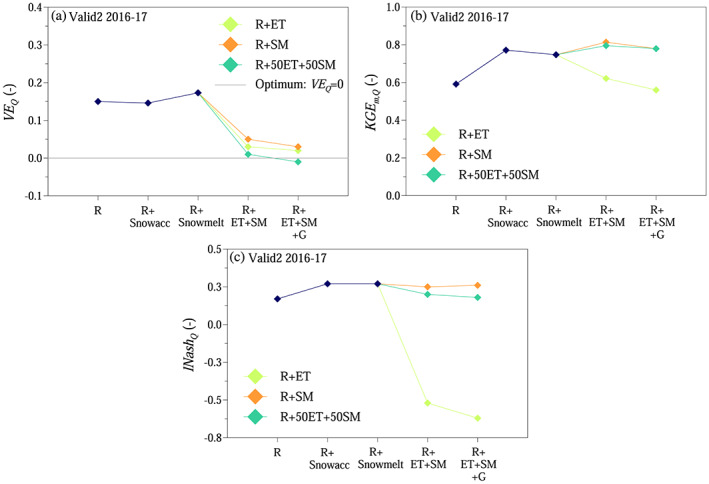
Runoff simulation efficiencies for each scenario during model validation (2016–2017): (a) relative volume error *VE*_*Q*_. (b) Monthly Kling‐Gupta efficiency *KGE*_*Q,m*_. (c) Daily logarithmic Nash Sutcliffe efficiency *lNash*_*Q*_. Scenarios according to Table 2.

**Figure 11:**
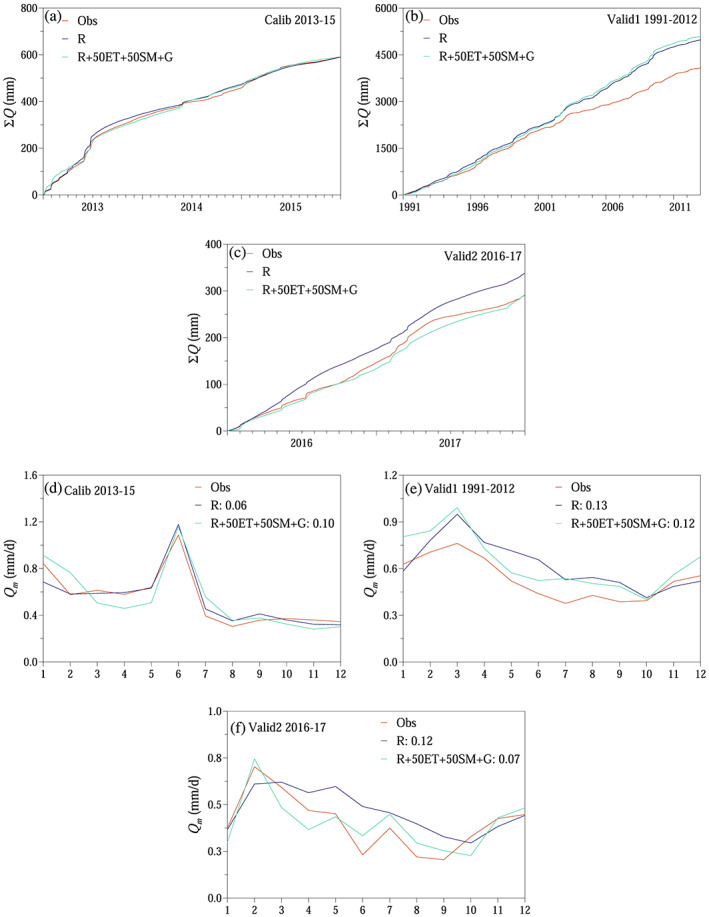
Runoff simulation: (a–c) cumulative runoff. (d–f) Monthly average runoff *Q*_*m*_. Scenarios according to Table 2. Numbers in legend indicate root mean squared error.

**Figure 12:**
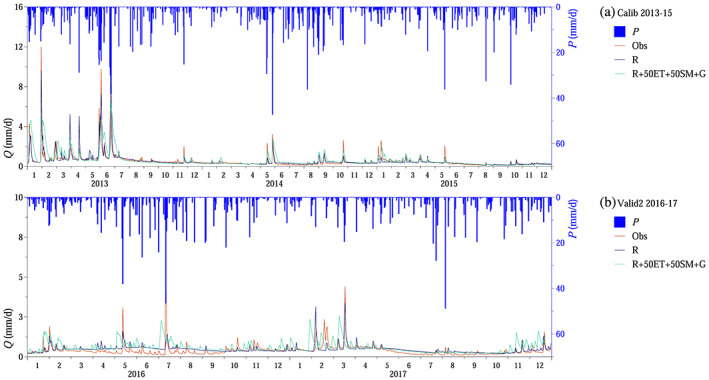
Runoff simulation: (a and b) daily runoff. Scenarios according to Table 2.

The final step was to evaluate the different scenarios in terms of simulating runoff on annual, seasonal, and daily time scales (Figures 10, 11, 12).

When comparing the evolution of the runoff simulation efficiencies through the different scenarios during the 2 year long validation period (2016–2017), it is clear that using additional data besides runoff for model calibration improved the runoff simulation efficiencies (Figure 10). The relative volume error slightly increased when snow data were also used for model calibration. But it definitely decreased, when soil moisture, evapotranspiration, overland flow, and storage change information was also used for model calibration. The monthly and daily runoff performance improved when additional data were also used for model calibration. Evapotranspiration and soil moisture data had the largest influence on runoff simulations, which also agreed with the results of the sensitivity analysis (Table S1). The sensitivity analysis showed that the most sensitive parameter belonged to the soil moisture accounting module. During the 2 year long validation period, the results improved the best if either both soil moisture and evapotranspiration or only soil moisture information was used during model calibration besides runoff.

On the annual time scale, the performance of the proposed step‐by‐step approach was similar to the performance of the traditional Scenario R, when only runoff was used for model calibration. The proposed method even outperformed Scenario R during the shorter, 2 year long validation period (Val2, 2016–2017). Runoff was overestimated during the longer, 22 year long validation period both by Scenario R and the proposed calibration approach (Figures 11a–11c).

Similarly, on the seasonal time scale, the proposed method could efficiently model runoff. The root mean squared error between simulated and observed monthly average runoff was lower during the validation periods (Figures 11d–11f).

On the daily time scale, the model performed worse during model calibration and the 22 year long validation, if additional data besides runoff were used for model calibration. The peaks were underestimated, and the recession was more delayed, when additional data were involved in the model calibration. But the daily performance slightly improved during the 2 year long validation period by using additional data and not only runoff (Figure 12).

## 5 Discussion

Actual field measurements are of utmost importance to understand and model catchment processes. In this study, we showed that by using a variety of different field observations, we were able to simulate not only runoff but also other hydrological processes generally efficiently using a lumped conceptual hydrologic model and a stepwise model calibration approach. This means that the model simulated runoff generally well for the right reasons (Grayson et al., 1992) on the annual and seasonal time scales. Avanzi et al. (2020) drew similar conclusions, when they found that ground‐based measurements allowed identifying more hydrologic model parameters than runoff alone. Rakovec et al. (2016) also noted that although it was necessary to constrain the model against runoff, this was not sufficient to simulate other variables, for instance, soil moisture, accurately.

In previous studies, which used additional data, not only runoff, to calibrate a hydrologic model, the simulation of certain state variables usually improved. But generally, these were only the targeted state variables, such as snow water equivalent only, or soil moisture only, or evapotranspiration only. For instance, Kundu et al. (2017) reported that using remotely sensed soil moisture improved the rainfall‐runoff response, but not the routing simulations. Rajib et al. (2016) also pointed out that using remotely sensed soil moisture improved the simulation of surface soil moisture, but runoff, evapotranspiration, and root zone soil moisture were less affected. In this study, we managed to improve snow accumulation, snowmelt, soil moisture, evapotranspiration, overland flow, and storage change simulations at the same time.

While the inclusion of data on state variables and fluxes can improve their simulations, this usually comes at a cost of slight deterioration of runoff simulations (e.g., Gui et al., 2019; Parajka et al., 2007; Rientjes et al., 2013; Seibert, 2000). Seibert (2000) explained this effect by problems with the model structure. When he modified the model structure of the model, the runoff simulation efficiencies increased. Gui et al. (2019) explained the deterioration of runoff simulations when including remotely sensed evapotranspiration in the calibration by a lack of emphasis on evapotranspiration in the model compared to runoff processes, and Rientjes et al. (2013) suggested that small errors in actual evapotranspiration can cause large errors in runoff due to the difference in the volume of water. Rientjes et al. (2013) also pointed out a mismatch between the definition of satellite‐based actual evapotranspiration estimates and the HBV‐based estimates. According to Parajka et al. (2007), “similar values of the runoff objective function do not necessarily imply a similar hydrological response of the catchment.” In the present study, the runoff calibration efficiency also deteriorated slightly when including additional data besides runoff, mainly on the daily time scale. The change was smaller during the validation periods. Moreover, we could improve runoff simulations during the second, 2 year long validation period, on all time scales, that is, annual, monthly, and daily. Overall, using additional information besides runoff gave more realistic parameter combinations (Table 4), and the simulation of model internal fluxes and states improved. The deterioration of runoff efficiencies during model calibration and the first validation periods can be explained by model structural errors or mismatches between the conceptual model and field observations, which are both discussed below.

The sensitivity of runoff simulations to changing model parameters depended on the module of the model, as shown by the sensitivity analysis (Table S1). Although parameterizing the snow module in this catchment is not as relevant as in an Alpine catchment due to the small amount of snow, the proposed method might be useful for other studies, where the precipitation partitioning is a crucial modeling step but usually lacks validation (e.g., Jennings et al., 2018). Parameters describing the soil moisture accounting routine of the model were the most sensitive according to the sensitivity analysis. Therefore, in this study observations of soil moisture and evapotranspiration played the most important role in parameter estimation. Using either both evapotranspiration and soil moisture or only soil moisture observations, the model's most sensitive routine could be parameterized well. Previous studies are mixed in terms of whether soil moisture and/or evapotranspiration observations improve the efficiency of runoff simulations. Nijzink et al. (2018) found that soil moisture satellite products were more effective than evaporation products for deriving more constrained parameter distributions. López et al. (2017) showed that estimating runoff was more efficient, if both soil moisture and evapotranspiration satellite products were involved in the model calibration. Bergström and Lindström (2015) argued that the relative importance of these observations of course depends on the time step of the model considering that evapotranspiration volumes are less than storage in the unsaturated zone on a daily basis. Similarly, Baroni et al. (2019) pointed out that different data collection strategies should be considered for different variables, depending on their degree of coupling to the atmosphere. Considering that changes in the atmosphere are faster, evapotranspiration observations are necessary on a daily basis, while changes in soil moisture and groundwater levels are more consistent over time, as these are more decoupled from the atmosphere.

Linking observations with model simulations is not a straightforward task, and local experience with the catchment processes may be a substantial advantage (Avanzi et al., 2020; Holländer et al., 2009). For physically based and spatially distributed models, modelers can often directly and explicitly compare measured and simulated volumes of water and energy fluxes (e.g., Kuras et al., 2011; Thyer et al., 2004). For conceptual hydrologic models, especially if the model is spatially lumped, a balance between the lumped conceptual model concept and spatial and temporal variability of processes has to be found. The spatial organization of soil moisture and other variables within the catchment may matter for runoff generation which cannot be captured by lumped models as they aggregate spatial processes variability (Blöschl et al., 1995; Viglione et al., 2018; Western et al., 1998). In this study, our aim was to use such objective functions and compare such quantities that helped bridging the gap between the model and observations. Therefore, instead of comparing volumes, we compared the standardized values of observed and simulated soil moisture and storage change. For the snow module, we used a binomial snow cover index based on different types of measurements to decide if there was snow in the catchment and a threshold for simulated snow water equivalent, only above which the catchment was considered snow covered. For the subsurface module, we compared the sign of the standardized storage change. When different objectives were tested instead of using these, the model often gave unrealistic results.

It is important to note that the aim of this study was not to modify or optimize the structure of the model but to use a conceptual model structure that has been used in various climatic regions in previous studies both in gauged and ungauged basins (e.g., Blöschl et al., 2013; Parajka et al., 2007; Sleziak et al., 2018), and that is general enough so that it could be used to simulate runoff in arbitrarily chosen basins. Previous studies proved that this type of model structure works well in Austrian catchments, both in Alpine and lowland environments (e.g., Parajka et al., 2007; Sleziak et al., 2018). Based on our current understanding on the HOAL, that is, how this catchment works conceptually, the HBV model structure is a very good representation of the catchment. Regarding the runoff generation in the catchment, the saturation excess overland flow events in the valley bottom identified by the weather station camera according to Silasari et al. (2017) could be well matched with the very fast runoff response of the HBV‐based TUWmodel. If a certain storage state threshold *LS*_*UZ*_ is reached in the upper reservoir of the model, that is, filling up from the bottom, which corresponds to saturation excess runoff mechanisms, very fast runoff starts. Regarding the subsurface mechanisms, Exner‐Kittridge et al. (2016) distinguished between two main aquifers in the HOAL, a shallow and a deep one. Their contribution to runoff depends on the hydrologic conditions, for example, low or high flow conditions, seasonality, and so forth. The runoff generation module of the HBV‐based TUWmodel consists of two subsurface reservoirs, the upper reservoir contributes to rainfall events, while the outflow from the lower reservoir takes place on much longer, monthly time scales.

Although we had a certain understanding how the catchment works conceptually, on small catchment scale, the role of inhomogeneous surface and subsurface properties (e.g., complex geology, cracks, and earthworm paths resulting in preferential flow paths) which result in nonlinear responses and processes with different thresholds (e.g., flow paths which are only activated above a certain groundwater level) can be more dominant than on large catchment scale where the response is averaged out. On small catchments, there are several exceptional cases, where the model does not work. The shorter the time scale, the more pronounced and visible these exceptions. The model in this study also underperformed on the daily time scale for simulating runoff during the validation periods. This underperfomance slightly improved during the second, 2 year long validation period where additional data were also used for model calibration. A possible reason for this daily underperformance for runoff could be that the model was trained on 3 years (2013–2015), which were extreme years including a very wet year (2013) and two very dry years (2014–2015). During extreme hydrologic conditions, that is, wet or dry years, the area contributing to runoff, the active flow paths, and the catchment rainfall response can be very different. Furthermore, the validation period was characterized by much more snow, while the calibration period was almost snow‐free. A lumped model often cannot handle these differences. Other reasons could be further structural errors of the model, for instance, in the evapotranspiration module. In the evapotranspiration module the simplified representation of actual evapotranspiration often does not match the measurements. During rainfall events, actual evapotranspiration measured by the eddy covariance systems drops to zero, while HBV‐based model simulations increase due to the higher soil moisture content. During the drier summer season, water for evapotranspiration might be extracted from deeper soil layers and not the surface layers monitored by the soil moisture sensors. A possible solution might be to use a distributed model, where a difference in model structure could better take into account the inhomogeneities of a small catchment, such as different runoff generation mechanisms, groundwater level‐dependent switches in flow paths. In this study we used only the lumped version of the model, which performed well on the annual and seasonal time scales. In order to improve the daily performance of the model, a spatial distribution and modified model structure might be necessary. A spatially distributed model structure may better take advantage of the higher information content of the measurement locations within the catchment. This suggests that an increased density of measurements that capture the spatial distribution of the variables may not necessarily benefit lumped model performance.

This study showed that by using field measurements of input and output fluxes and states besides runoff, we were able to simulate these fluxes and states (snow, sail moisture, evapotranspiration, and storage), moreover also the annual and seasonal runoff more efficiently. This finding suggests that hydrologic models which are constrained only by runoff might be simulating runoff well for the wrong reasons, that is, with wrong parameters. But this can be only revealed if other state variables of the model are tested against observations. For small basins, where satellite information may be too coarse, field observations play a crucial role. For instance, if data on evapotranspiration, soil moisture, or saturation areas are available, these may be complementary to existing runoff data or surrogates of runoff data if no runoff measurements exist. They can all help in constraining a hydrologic model.

## 6 Conclusions

This study presented a new framework for estimating the parameters of runoff model components in a stepwise fashion from field observations of input and output fluxes and states besides runoff and investigated the value of these data for simulating runoff well for the right reasons in a small agricultural catchment. Our results showed the following:

1. By using the proposed step‐by‐step model calibration approach with different field observations of input and output fluxes and states besides runoff for parameter estimation, we were able to efficiently simulate these fluxes and states correctly. This means that we simulated runoff well for the right reasons on the annual and seasonal time scales.
2. For this catchment, field observations of soil moisture and evapotranspiration had the largest influence on runoff simulations.
3. Future research and possibly a spatially distributed and modified model structure might be necessary on a small catchment to take into account the role of small‐scale inhomogeneities and to improve the daily performance of the model. Distributed and more physically based models may better reflect the catchment dynamics and may be more compatible with the spatially distributed measured dynamics.

## Supporting information

Supporting Information S1

Supporting Information S2

Table S1

## Data Availability

The data used are listed in the tables, and the data necessary to reproduce the reported findings are available on the following site: https://owncloud.tuwien.ac.at/index.php/s/uQfz97O14srsW50 (password: 20cWRs19).
