# Supplementary material for: The Added Value of Different Data Types for Calibrating and Testing a Hydrologic Model in a Small Catchment
Source: Water Resour Res. 2020 Oct 8;56(10):e2019WR026153. doi: 10.1029/2019WR026153 (PMC7594447; doi:10.1029/2019WR026153)
Supplement: Supplementary file 1 — Supporting Information S1 [file WRCR-56-e2019WR026153-s001.docx]

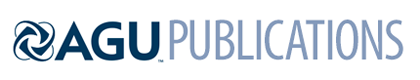


*Water Resources Research*

Supporting Information for

**The added value of different data types for calibrating and testing a hydrologic model**

B. Széles^1^, J. Parajka^1^, P. Hogan^1^, R. Silasari^1^, L. Pavlin^1^, P. Strauss^2^, and G. Blöschl^1^

^1^Institute of Hydraulic Engineering and Water Resources Management, Vienna University of Technology, Karlsplatz 13/222, 1040 Vienna, Austria

^2^Federal Agency of Water Management, Institute for Land and Water Management Research, Pollnbergstraße 1, 3252 Petzenkirchen, Austria

**Contents of this file**

Text S1

**Introduction**

Text1 contains details on the sensitivity analysis.

Text S1. Sensitivity analysis

The influence of changes in the model parameters on the daily runoff hydrograph was assessed by the LH-OAT method, a global sensitivity analysis method (van Griensven & Meixner, 2003; van Griensven et al., 2006), which combines the Latin-Hypercube (McKay et al., 1979) with the One-Factor-At-a-Time sampling (Morris, 1991). The number of intervals in the Latin Hypercube was chosen as 500 and the parameter change for OAT fraction as 0.05. The changes in the runoff hydrograph relative to the base case (LH point) was measured by the Nash-Sutcliffe coefficient. The mean sensitivities of the 14 model parameters were compared.

Comparing the mean sensitivities of the model parameters assessed by the daily Nash Sutcliffe coefficient for runoff, the two most sensitive parameters were the field capacity *FC* and the storage time for very fast response *k_0_* (Supporting Information Table S2). The same parameters were found to be the most sensitive for all time periods (2013-15 calibration period in Table S2, 1991-2012 and 2016-17 validation periods not shown here).
