# Supplementary material for: The Added Value of Different Data Types for Calibrating and Testing a Hydrologic Model in a Small Catchment
Source: Water Resour Res. 2020 Oct 8;56(10):e2019WR026153. doi: 10.1029/2019WR026153 (PMC7594447; doi:10.1029/2019WR026153)
Supplement: Supplementary file 2 — Supporting Information S2 [file WRCR-56-e2019WR026153-s002.docx]

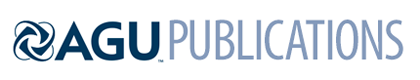


*Water Resources Research*

Supporting Information for

**The added value of different data types for calibrating and testing a hydrologic model**

B. Széles^1^, J. Parajka^1^, P. Hogan^1^, R. Silasari^1^, L. Pavlin^1^, P. Strauss^2^, and G. Blöschl^1^

^1^Institute of Hydraulic Engineering and Water Resources Management, Vienna University of Technology, Karlsplatz 13/222, 1040 Vienna, Austria

^2^Federal Agency of Water Management, Institute for Land and Water Management Research, Pollnbergstraße 1, 3252 Petzenkirchen, Austria

**Contents of this file**

Text S2

**Introduction**

Text2 contains details on the calculation of the wet bulb temperature during preceipitation events with a shift in precipitation phase.

Text S2. Wet bulb temperature

Those precipitation events were selected during the model calibration period (2013-15), when a shift was observed in the phase of the precipitation measured by the Present Weather Sensor (from snow to rain, i.e. from category 3 to 2, or from rain to snow, i.e. from category 2 to 3). The wet bulb temperature was calculated with the R package bigleaf (Knauer et al., 2018) for each two half hourly time steps during the precipitation phase shifts (one half hour with category 3, and the following half hour with category 2, or vice versa). The average of the wet bulb temperature for each two half hourly time steps was calculated. And finally, the median of the average wet bulb temperature was extracted.
