# Supplementary material for: The Added Value of Different Data Types for Calibrating and Testing a Hydrologic Model in a Small Catchment
Source: Water Resour Res. 2020 Oct 8;56(10):e2019WR026153. doi: 10.1029/2019WR026153 (PMC7594447; doi:10.1029/2019WR026153)
Supplement: Supplementary file 3 — Table S1 [file WRCR-56-e2019WR026153-s003.docx]

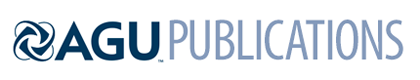


*Water Resources Research*

Supporting Information for

**The added value of different data types for calibrating and testing a hydrologic model**

B. Széles^1^, J. Parajka^1^, P. Hogan^1^, R. Silasari^1^, L. Pavlin^1^, P. Strauss^2^, and G. Blöschl^1^

^1^Institute of Hydraulic Engineering and Water Resources Management, Vienna University of Technology, Karlsplatz 13/222, 1040 Vienna, Austria

^2^Federal Agency of Water Management, Institute for Land and Water Management Research, Pollnbergstraße 1, 3252 Petzenkirchen, Austria

**Contents of this file**

Table S1

**Introduction**

Table S1 contains the results of the sensitivity analysis.

| Module | Parameter (unit) | Mean sensitivity (%) |
| --- | --- | --- |
| Snow | *SCF* (-) | 0.7 |
|  | *DDF* (mm/°C/d) | 5.1 |
|  | *T_wb_* (°C) | 0.7 |
|  | *T_m_* (°C) | 1.8 |
| Soil moisture accounting | *LP_rat_* (-) | 2.4 |
|  | *FC* (mm) | 37.7 |
|  | *β* (-) | 6.2 |
| Runoff generation | *k_0_* (d) | 18.6 |
|  | *k_1_* (d) | 8.2 |
|  | *k_2_* (d) | 0.6 |
|  | *LS_UZ_* (mm) | 0.6 |
|  | *c_P_* (mm/d) | 11.2 |
|  | *B_MAX_* (d) | 1.7 |
|  | *c_R_* (d^2^/mm) | 4.4 |

Table S1. Results of the LH-OAT sensitivity analysis for the calibration period 2013-15: mean sensitivities (%) of 14 free parameters, assessed by the influence of model parameters on the change in daily Nash-Sutcliffe coefficient for runoff. The most sensitive model parameter is *FC* (field capacity) of the soil moisture accounting module as would be expected in a humid climate.
